# Functional and genomic characterization of patient‐derived xenograft model to study the adaptation to mTORC1 inhibitor in clear cell renal cell carcinoma

**DOI:** 10.1002/cam4.3578

**Published:** 2020-10-27

**Authors:** Hiromasa Sakamoto, Toshinari Yamasaki, Takayuki Sumiyoshi, Masashi Takeda, Noboru Shibasaki, Noriaki Utsunomiya, Ryuichiro Arakaki, Shusuke Akamatsu, Takashi Kobayashi, Takahiro Inoue, Tomomi Kamba, Eijiro Nakamura, Osamu Ogawa

**Affiliations:** ^1^ Department of Urology Kyoto University Graduate School of Medicine Kyoto Japan; ^2^ Department of Nephro‐Urologic Surgery and Andrology Mie University Graduate School of Medicine Tsu Japan; ^3^ Department of Urology Kumamoto University Graduate School of Medical Sciences Kumamoto Japan; ^4^ DSK Project, Medical Innovation Center Kyoto University Graduate School of Medicine Kyoto Japan

**Keywords:** DNMT1, drug resistance, methylation, mTOR, renal cell carcinoma

## Abstract

Resistance to the mechanistic target of rapamycin (mTOR) inhibitors, which are a standard treatment for advanced clear cell renal cell carcinoma (ccRCC), eventually develops in most cases. In this study, we established a patient‐derived xenograft (PDX) model which acquired resistance to the mTOR inhibitor temsirolimus, and explored the underlying mechanisms of resistance acquisition. Temsirolimus was administered to PDX model mice, and one cohort of PDX models acquired resistance after repeated passages. PDX tumors were genetically analyzed by whole‐exome sequencing and detected several genetic alterations specific to resistant tumors. Among them, mutations in *ANKRD12* and *DNMT1* were already identified in the early passage of a resistant PDX model, and we focused on a *DNMT1* mutation as a potential candidate for developing the resistant phenotype. While DNMT1 expression in temsirolimus‐resistant tumors was comparable with the control tumors, DNMT enzyme activity was decreased in resistant tumors compared with controls. Clustered regularly interspaced short palindromic repeats (CRISPR)/CRISPR‐associated protein 9‐mediated heterozygous knockdown of DNMT1 in the temsirolimus‐sensitive ccRCC (786‐O) cell line was shown to result in a temsirolimus‐resistant phenotype in vitro and in vivo. Integrated gene profiles using methylation and microarray analyses of PDX tumors suggested a global shift for the hypomethylation status including promotor regions, and showed the upregulation of several molecules that regulate the mTOR pathway in temsirolimus‐resistant tumors. Present study showed the feasibility of PDX model to explore the mechanisms of mTOR resistance acquisition and suggested that genetic alterations, including that of *DNMT1*, which alter the methylation status in cancer cells, are one of the potential mechanisms of developing resistance to temsirolimus.

## Graphical abstract

This study showed the feasibility of PDX model to explore the mechanisms of mTOR resistance acquisition and suggested that genetic alterations, including that of DNMT1, which alter the methylation status in cancer cells, are one of the potential mechanisms of developing resistance to temsirolimus.

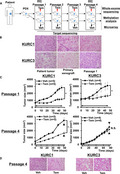

## 1 INTRODUCTION

Clear cell renal cell carcinoma (ccRCC) accounts for approximately 3% of all adult malignancies, and around 15% are metastatic at diagnosis.^1^ Several drugs such as tyrosine kinase inhibitors, programmed death 1 checkpoint inhibitors, and mechanistic target of rapamycin (mTOR) inhibitors have been used for the treatment of metastatic ccRCC.^2^

Mechanistic target of rapamycin is a serine threonine kinase which forms the subunit of two multi‐protein complexes, mTOR complex 1 (mTORC1) and mTOR complex 2 (mTORC2). mTORC1 controls the cell growth and metabolism, while mTORC2 regulates the cell proliferation and survival.^3^ Rapamycin forms a complex with 12‐kD FK506 binding protein (FKBP12), leading to the inhibition of mTORC1. Temsirolimus is an example of an mTORC1 inhibitor, like rapamycin, that has improved the prognosis of ccRCC patients.^4^ Temsirolimus was also approved for use in metastatic ccRCC patients with poor prognosis.^5^ However, despite its efficacy, ccRCC often develops resistance to temsirolimus and clinically shows progression within several months after treatment initiation.

Several mechanisms for acquired resistance to temsirolimus have been described, including mutations in mTOR, activation of an alternative signaling pathway, and intratumoral heterogeneity.^6^, ^7^ A previous report showed that mTOR mutations block the binding of rapamycin‐FKBP12 to mTOR, resulting in rapamycin resistance.^8^ Other reports suggested that the suppression of negative feedback loops leads to the activation of phosphoinositide 3‐kinase (PI3K)/AKT and RAS/RAF/MEK/mitogen‐activated protein kinase (MAPK) pathways which impede the efficacy of rapamycin.^9^, ^10^ Moreover, multiregional sequencing revealed the existence of intratumoral heterogeneity in ccRCC.^11^ This is thought to be related to the heterogeneous function of mTORC1 activity, possibly leading to differences in sensitivities to mTORC1 inhibitors.^8^ However, this has not yet been fully elucidated.

Patient‐derived xenograft (PDX) models have been a useful platform for drug screening, biomarker development, predictions of drug responses, and the analysis of drug resistance.^12^, ^13^ Moreover, PDX tumors maintain the histological and genomic profiling of primary tumors.^14^, ^15^ Several ccRCC PDX models have been used to evaluate drug resistance,^16^, ^17^ and we previously used such a model to show that interleukin 13 receptor subunit alpha 2 mediates ccRCC sunitinib resistance.^18^ However, few studies have investigated temsirolimus resistance using ccRCC‐PDX models.^19^

Therefore, in the present study, we aimed to clarify the genetic mechanisms underlying resistance to temsirolimus using PDX models and to identify potential targets to overcome this resistance.

## 2 MATERIALS AND METHODS

### 2.1 Patients and ccRCC samples

Tumor specimens were obtained from the Department of Urology, Kyoto University Hospital with appropriate informed consent under approval by Kyoto University's Institutional Review Board (IRB approval number G52, G504).

### 2.2 Establishment of xenograft models

All experiments with laboratory animals were performed in accordance with Guidelines for Animal Experiments of Kyoto University (Permit Number 15288). To establish a PDX model, patient tumors were minced into 20–30 mm^3^ fragments, and subcutaneously transplanted into 5‐week‐old CB‐17/Icr‐crj severe combined immunodeficiency (SCID) mice (Charles River, Yokohama, Japan) on the day of surgery. Tumor size was measured weekly, and when tumors reached 2000–3000 mm^3^, they were removed, split, and re‐transplanted in the same manner. We kept three PDX model cohorts (KURC1, 2, and 3). To establish cell line xenograft models, a total of 1.0 × 10^7^ cells were subcutaneously injected into 5–6‐week‐old female BALB/cA Jcl nude (nu/nu) mice (CLEA, Tokyo, Japan). All experiments were performed under sodium pentobarbital anesthesia, and all efforts were made to minimize suffering. Following the experimental procedures, all animals were euthanized by carbon dioxide and tumors were excised.

### 2.3 In vivo temsirolimus administration

**FIGURE 1:**
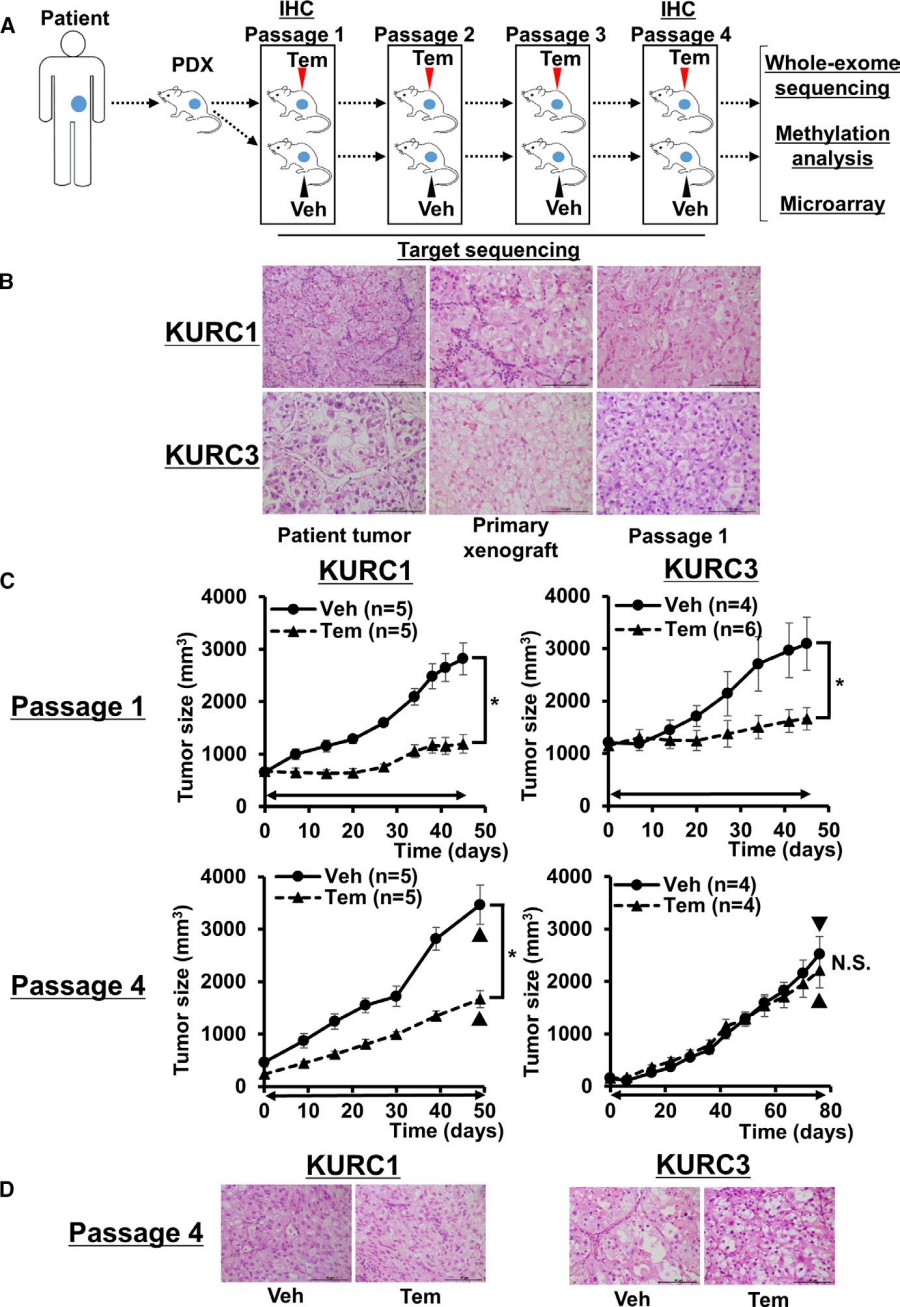
(A) Strategy of the generation of temsirolimus‐resistant PDX models. IHC, immunohistochemistry; Tem, temsirolimus; Veh, vehicle. (B) Hematoxylin and eosin (H&E) staining shows patient tumor, primary xenograft tumor, and the tumor of passage 1 in KURC1 and KURC3. Scale bar, 100 μm. (C) Sequential changes of KURC1 or KURC3 xenograft tumors (passage 1 and passage 4 treated with vehicle or temsirolimus). Each time point represents the mean ± SE of tumor volume in each group. Day 0 is the administration day. Arrowed bars indicate the periods of temsirolimus administration. ▼ indicates the time point when tumors were resected for whole‐exome sequencing. Statistical analysis was performed using two‐way repeated ANOVA (*p < 0.05, NS, not significant). Tem, temsirolimus; Veh, vehicle. (D) H&E staining shows tumors of passage 4 in KURC1 and KURC3 treated with vehicle or temsirolimus. Scale bar, 100 μm

Temsirolimus (Pfizer Global Pharmaceuticals) was diluted as previously described^20^ and used at a previously determined dose in RCC or other type of cancer.^21^, ^22^ Ten mg/kg temsirolimus or vehicle only was intraperitoneally administered once a week. Treatment with temsirolimus or vehicle was started simultaneously in each cohort at 6‐week intervals after tumor inoculation in passage 1 and at 2‐week intervals after tumor inoculation in passages 2–4. In passage 1 cohorts, administration was started after the tumor diameter was increased enough to clearly confirm the initial antitumor effect of temsirolimus, but in passage 2–4 cohorts, the drug was planned to start administration 2 weeks interval from tumor transplantation in order to accelerate the selection of cells that acquired resistance. Tumors were considered resistant when their growth rate was equal to that of control tumors. Once xenograft tumors had developed sufficiently, they were removed and transplanted into other SCID mice. Two weeks later, temsirolimus was re‐administered to these mice, and this was repeated until tumors became resistant to temsirolimus (Figure 1A; Table S1). KURC1 PDX tumors treated with vehicle or temsirolimus in passage 1 were named KURC1 Veh/P1 and Tem/P1, respectively. Other KURC PDX tumors in each passage were named in the same manner.

### 2.4 Whole‐exome sequencing

Whole‐exome sequencing (WES) analysis was performed on one KURC1 primary tumor, three KURC1 PDX tumors (KURC1 Veh/P4/#1, Tem/P4/#1, and #2), and three KURC3 PDX tumors (KURC3 Veh/P4/#1, Tem/P4/#1, and #2) using the Agilent SureSelect All Exon V5 capture (Agilent Technologies) and paired‐end (100 bp) sequencing on an HiSeq 2500 sequencing system (Illumina Inc.) at Hokkaido System Science Co., Ltd. (Table S1). Sequencing reads were aligned to the human genome GRCh37 (hg19) and mouse genome GRCm38 (mm10) to remove mouse stroma reads from human tumor reads using Burrows–Wheeler Aligner version 0.7.10 (http://bio‐bwa.sourceforge.net/). Sequence realignment was performed with SAMtools version 1.2 (http://www.htslib.org/) and The Genome Analysis Toolkit lite version 2.3.0 (https://www.broadinstitute.org/gatk/). PCR duplicates were removed with Picard version 1.133 (http://broadinstitute.github.io/picard/). Variants were called using SAMtools version 1.2 and BCFtools version 1.2 (http://www.htslib.org/), and were annotated by SnpEff version 4.1 (http://snpeff.sourceforge.net/index.html/).^23^ Variants with a coverage greater than ×10 and with a Phred quality score greater than 20 were filtered. Variants assigned to the single nucleotide polymorphism database, those that were synonymous, and intron variations were filtered out. A comparison of temsirolimus and vehicle data identified variants with an allele frequency change of ≥35%. The impact of single nucleotide variants (SNVs) was predicted by FATHMM‐XF.^24^ Our WES data were registered at the NCBI SRA (PRJNA552443).

### 2.5 DNA methyltransferase activity/inhibition assay

DNMT enzyme activity of cell lines and xenograft tumors was evaluated using the DNMT Activity/Inhibition Assay (Active Motif) according to the manufacturer's instructions. Nuclear extracts were prepared by the Nuclear Extract Kit (Active Motif). Each 10 μg of nuclear extract was incubated with the enzymatic buffer containing AdoMet (1:100 dilution) for 2 h, and incubated with His‐MBD2b protein (1:50 dilution) for 45 min. followed by incubation for 45 min with anti‐polyHis‐HRP antibody (1:1000 dilution). The developing solution was added to each extract and the optical density was measured on a microplate reader at 450 nm.^25^

### 2.6 Clustered regularly interspaced short palindromic repeats (CRISPR)/CRISPR‐associated protein 9 (Cas9)‐mediated gene editing

DNMT1 editing was performed using the CRISPR/Cas9 system. Guide (g)RNAs directed against DNMT1 were cloned into the pSpCas9(BB)‐2A‐GFP(PX458) vector, which was a gift from Feng Zhang (Addgene plasmid #48138).^26^ The RNA sequences were 5′‐GCTTTTCGCGCGGAAACCGA‐3′ (upstream) and 5′‐CGACGATGTCCGCAGGCGGT‐3′ (downstream). 786‐O cells were transfected with the gRNA‐expressing plasmid using Lipofectamine 2000 (Thermo Fisher Scientific) according to the manufacturer's protocol. Two days later, single clone green fluorescent protein (GFP)‐positive cells were sorted by flow cytometry on the FACSAria2 flow cytometer (BD Biosciences). To sort single cells into individual wells, 150 cells of GFP‐positive clones were transferred to three 96‐well plates. Single cell clones with heterozygous knockdown of the target region were selected for further analysis by PCR.

### 2.7 DNA methylation analysis

DNA methylation profiling was performed for two KURC3 PDX tumors (Veh/P4/#1 and Tem/P4/#1) at Takara Bio Co., Ltd. using the Infinium HumanMethylation450 BeadChip array (Illumina Inc.) (Table S1). Data processing was performed using Illumina GenomeStudio V2011.1 and Methylation Module version 1.9.0. Signal intensities were obtained with background subtraction and normalized to internal controls. Beta‐values were normalized according to the peak‐based correction method using R version 3.1.0 (R Development Core Team, 2015).^27^ A comparison of vehicle and temsirolimus data identified hypermethylation or hypomethylation probes with a change of ≥1.5‐fold. Our methylation data were registered at the NCBI GEO (GSE133444).

### 2.8 Microarray analyses

Microarray analyses were performed for KURC1 PDX tumors (Veh/P4/#1, Tem/P4/#1, and #2) and KURC3 PDX tumors (Veh/P4/#1 and #2, Tem/P4/#1, #2, #3, and #4) at Genetic Lab Co., Ltd. using the GeneChip Human Gene 2.0 ST Array (Affymetrix) (Table S1). Data were analyzed using GeneSpring GX ver. 12.6 (Agilent Technologies). A comparison of vehicle and temsirolimus data identified upregulated or downregulated probes with a fold‐change of ≥1.2. Our microarray data were registered at the NCBI GEO (GSE133446).

### 2.9 TCGA data analyses using cBioPortal

TCGA data were accessed via cBioPortal in August–September 2019; provisional datasets for kidney renal clear cell carcinoma were analyzed. All statistical analyses were derived from cBioPortal tools.^28^, ^29^

### 2.10 Human Protein Atlas analyses

The Human Protein Atlas explores the clinical outcome of each protein‐coding gene in 17 different cancers including kidney cancers. All data presented are available from www.proteinatlas.org.^30^

### 2.11 Statistical analysis

Data are expressed as the mean ± SE. Differences between means and microarray analysis findings were analyzed using the Student's *t*‐test. Tumor growth in vitro and in vivo was assessed by two‐way repeated analysis of variance (ANOVA). *p* values <0.05 were considered statistically significant.

## 3 RESULTS

### 3.1 Establishment and characterization of the primary RCC xenograft model

Three cohorts of primary xenografts, KURC1, 2, and 3, were already established and stably engrafted following three or more passages in vivo.^18^, ^31^ The histology and clinical stage of these patient tumors were: KURC1, primary tumor of clear cell‐type RCC of Grade 2–3 pT2N0M0; KURC2, primary tumor of clear cell‐type RCC of Grade 3 pT3aN0M1; and KURC3, skin metastasis of clear cell‐type RCC Grade 3 pT3bN2M1 (Figure 1A). The growth of both KURC1 and KURC3 PDX tumors treated with sunitinib was initially suppressed but regrowth gradually occurred, while KURC2 PDX tumor growth was suppressed for a long period (Figure S1).^18^

Considering that mTORC1 inhibitors are often administered to patients who show tyrosine kinase inhibitor resistance, we focused on KURC1 and KURC3 PDX models which had potentially decreased sensitivity to sunitinib. Both xenograft models had mostly recaptured the histopathological features of the original tumors in terms of tumor grade and architecture (Figure 1B). WES was used to identify mutations related to ccRCC in these tumors. *SETD2* (c.5015+3A>C), *ARID1A* (p.Glu2250fs), and *SLC27A6* (c.1165‐5dupT) mutations and *VHL* (p.Phe76del), *BAP1* (p.Pro555fs), and *TP53* (p.Ala276Asp) mutations ware identified in KURC1 and KURC3 xenograft tumors, respectively.

### 3.2 Response and acquisition of resistance to temsirolimus in a primary xenograft model

To clarify the response to the mTOR inhibitor in these xenograft models, temsirolimus or vehicle treatment was started. In the first passage after temsirolimus administration, KURC1 Tem/P1 and KURC3 Tem/P1 tumor growth was suppressed for around 50 days (Figure 1C). Both tumors then sufficiently increased in size, and were transferred to other SCID mice for re‐treatment with temsirolimus (Figure 1A). In the next passage, KURC3 Tem/P2 was shown to regrow even after temsirolimus administration, while KURC1 Tem/P2 growth remained suppressed (Figure S2). After three passages, KURC3 Tem/P4 tumors exhibited a similar tumor growth curve to KURC3 Veh/P4 tumors (Figure 1C), and maintained their clear‐cell histological appearance (Figure 1D). These findings suggest that KURC1 PDX tumors retained sensitivity while KURC3 PDX tumors developed resistance to temsirolimus.

### 3.3 WES revealed several gene alterations in temsirolimus‐resistant PDX tumors

Next, we explored the mechanisms of temsirolimus resistance acquisition using these established PDX models (Figure 1A). Initially, to elucidate the genomic changes harbored by temsirolimus‐resistant tumors, we performed WES for the KURC1 primary tumor, three cohorts of KURC1 PDX tumors (KURC1 Veh/P4/#1, Tem/P4/#1, and #2), and three cohorts of KURC3 PDX tumors (KURC3 Veh/P4/#1, Tem/P4/#1, and #2) (Table S1). Sequence data of PDX tumors showed that 16%–35% of reads were aligned to the mouse genome, while only 0.09% of reads were non‐specifically aligned to the mouse genome in the primary tumor. The mean target coverage across the exomes, which were aligned to the human genome, varied from ×37 to 52, and 92% of the target bases had a coverage of ×10 (Table S2).

**FIGURE 2:**
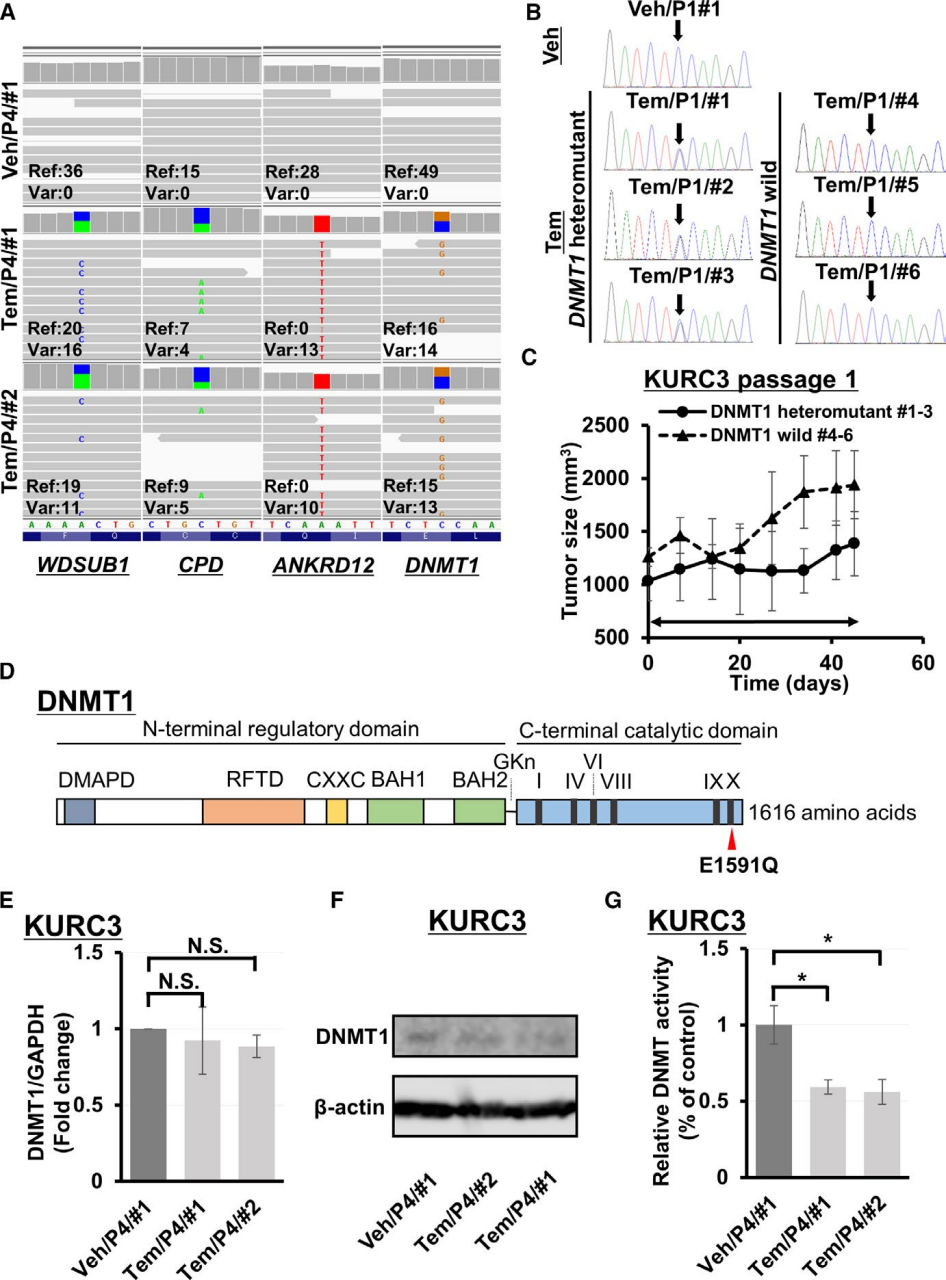
(A) Integrated Genomic Viewer shows *WDSUB1* c.313A>C, *CPD* c.3552C>A, *ANKRD12* c.2481A>T, and *DNMT1* c.4771G>C mutations in KURC3 passage 4 tumors treated with vehicle or temsirolimus. P4, passage 4; Ref, reference; Tem, temsirolimus; Var, variant; Veh, vehicle. (B) Sanger sequencing shows a heterozygous *DNMT1* missense mutation in Tem/P1/#1, #2, and #3, but not in Tem/P1/#4, #5, #6, or Veh/P1/#1. (C) Sequential changes of subcutaneous xenograft tumors from *DNMT1* heteromutant tumors (Tem/P1/#1, #2, and #3) or *DNMT1* wild‐type tumors (Tem/P1/#4, #5, #6) treated with temsirolimus. Day 0 is the administration day. Arrowed bars indicate the periods of temsirolimus administration. Each time point represents the mean ± SE of tumor volume in each group. (D) Schematic of DNMT1 protein. DMAPD, DNA methyltransferas‐associated protein 1 interacting domain (amino acids 18–103); RFTD, Replication foci targeting domain (amino acids 350–600); CXXC, CXXC domain (amino acids 650–699); BAH1 and BAH2, bromo‐adjacent homology domains 1 and 2 (amino acids 755–880 and 972–1100); GKn, glycine lysine repeats; I, IV, VI, VIII, IX, and X, conserved C5 DNA MTase motifs I, IV, VI, VIII, IX, and X in the C‐terminal part. (E) Evaluation of DNMT1 mRNA expression in KURC3 passage 4 xenograft tumors treated with vehicle or temsirolimus by quantitative PCR. All samples were prepared in triplicate and data are presented as the mean ± SE. Columns, mean; bar, SE. There was no significant difference in each group (NS, not significant; Student's *t*‐test). (F) Evaluation of DNMT1 protein expression in KURC3 passage 4 xenograft tumors treated with vehicle or temsirolimus by immunoblotting. (G) Evaluation of DNMT activity in KURC3 passage 4 xenograft tumors treated with vehicle or temsirolimus by the DNMT Activity/Inhibition Assay. All samples were prepared in triplicate and data are presented as the mean ± SE. Columns, mean; bar, SE. The difference in DNMT activity between tumors treated with vehicle or temsirolimus in KURC3 was statistically significant (*p < 0.05; Student's *t*‐test)

Eleven and 10 somatic variants were identified in KURC3 Tem/P4/#1‐ and Tem/P4/#2‐ resistant tumors, respectively (Table 1). Four variants, *WDSUB1*, *CPD*, *ANKRD12*, and *DNMT1*, were common between two individual tumors of KURC3 Tem/P4 (Table 1; Figure 2A). These variants were not identified in the KURC1 primary tumor or KURC1 PDX tumors (Table S3). To evaluate when these variants occurred, Sanger sequencing was performed for KURC3 PDX tumors of each generation. This revealed that *ANKRD12* and *DNMT1* variants were already identified in KURC3 Tem/P1 tumors, while *WDSUB1* and *CPD* variants were identified in KURC3 Tem/P2 tumors and subsequent passages (Table 2; Figure S3). Considering that KURC3 Tem/P2 tumors showed acquired resistance to temsirolimus (Figure S2), *DNMT1* and *ANKRD12* variants appear to be more involved in resistance. The impact prediction of SNVs by FATHMM‐XF suggested that only the *DNMT1* variant was potentially pathogenic, while the other three variants, in *ANKRD12*, *WDSUB1*, and *CPD*, were not (Table 1). Therefore, we focused on the *DNMT1* variant to determine whether it was associated with temsirolimus resistance.

**TABLE 1:** The variants identified in KURC3 Tem/P4/#1, #2 tumors

Gene	Chr	Start position	Ensemble	Allele change	Mutation type	Allele frequency (Tem)	Allele frequency (Veh)	Amino acid change	FATHMM prediction
P4/tem/#1									
***WDSUB1***	**2**	**160139268**	**‘00000359774**	**c.313A>C**	**Missense**	**0.444**	**0**	**p.Phe105Val**	**Benign (score 0.23)**
*HTR1A*	5	63257267	‘00000323865	c.280C>T	Missense	0.545	0	p.Ala94Thr	Pathogenic (score 0.60)
*FTMT*	5	121187900	‘00000321339	c.242A>C	Missense	0.393	0	p.Asn81Thr	Pathogenic (score 0.66)
*PCDHB12*	5	140591063	‘00000239450	c.*196 T>G	3′ UTR	0.428	0		Benign (score 0.31)
*RIC8A*	11	209894	‘00000325207	c.624_626delCCC	Inframe deletion	0.733	0.352	p.Pro209del	N/A
*QSER1*	11	32953288	‘00000399302	c.98‐1G>C	Splice acceptor	0.45	0		Pathogenic (score 0.99)
*DLG2*	11	83197206	‘00000530800	c.*60_*61insATCA	3′ UTR	0.681	0.25		N/A
***CPD***	**17**	**28788251**	**‘00000225719**	**c.3552C>A**	**Nonsense**	**0.363**	**0**	p.Cys1184*	**Benign (score 0.14)**
***ANKRD12***	**18**	**9255746**	**‘00000262126**	**c.2481A>T**	**Missense**	**1**	**0**	**p.Gln827His**	**Benign (score 0.22)**
***DNMT1***	**19**	**10244938**	**‘00000340748**	**c.4771C>G**	**Missense**	**0.466**	**0**	**p.Glu1591Gln**	**Pathogenic (score 0.85)**
*ATP5L2*	22	43035888	‘00000505920	c.*90A>T	3′ UTR	0.363	0		Pathogenic (score 0.68)
P4/tem/#2									
***WDSUB1***	**2**	**160139268**	**‘00000359774**	**c.313A>C**	**Missense**	**0.366**	**0**	**p.Phe105Val**	**Benign (score 0.23)**
*SCRN3*	2	175292580	‘00000272732	c.1251_1263delTTTATCAGTCAAA	frameshift	0.75	0.235	p.Asn417 fs	N/A
*FAM208B*	10	5805087	‘00000328090	c.*94_*95delCA	3′ UTR	0.785	0.375		N/A
*GALNT4*	12	89916794	‘00000529983	c.1525_1526insA	Frameshift	0.545	0	p.Lys510 fs	N/A
*SSH1*	12	109192834	‘00000326495	c.1291C>T	Missense	0.615	0.235	p.Arg431Cys	Benign (score 0.37)
*HNF1A*	12	121434637	‘00000402929	c.1408_1409insTTTCATTC	Frameshift	0.812	0.333	p.His470fs	N/A
***CPD***	**17**	**28788251**	**‘00000225719**	**c.3552C>A**	**Nonsense**	**0.357**	**0**	p.Cys1184*	**Benign (score 0.14)**
***ANKRD12***	**18**	**9255746**	**‘00000262126**	**c.2481A>T**	**Missense**	**1**	**0**	**p.Gln827His**	**Benign (score 0.22)**
***DNMT1***	**19**	**10244938**	**‘00000340748**	**c.4771C>G**	**Missense**	**0.464**	**0**	**p.Glu1591Gln**	**Pathogenic (score 0.85)**
*KIAA0930*	22	45607974	‘00000251993	c.94C>A	Missense	0.372	0	p.Asp32Tyr	Pathogenic (score 0.95)

Note

The common gene alterations between two tumors (Tem/P4/#1 and #2) were shown by bold letters.

Abbreviations: Chr, chromosome; N/A, not applicable; P4, passage 4; Tem, temsirolimus; Veh, vehicle.

**TABLE 2:** Mutation status of each generation in KURC3 Tem tumors

Gene	P1	P2	P3
*ANKRD12*	+/−	−/−	−/−
*DNMT1*	+/−	+/−	+/−
*WDSUB1*	+/+	+/−	+/−
*CPD*	+/+	+/−	+/−

Abbreviations: −/−, homozygous mutation; +/−, heterozygous mutation; +/+, wild; P1, passage 1; Tem, temsirolimus.

### 3.4 Temsirolimus‐resistant PDX tumors harbored a *DNMT1* variant and showed decreased DNMT enzyme activity

The *DNMT1* variant was identified in three of six KURC3 Tem/P1 tumors (Figure 2B). Growth of these tumors was initially suppressed but regrowth gradually occurred (Figure 2C). The missense variant c.4771G>C (p.Glu1591Gln) is located in the conserved C5 DNA MTase motif X of the C‐terminal catalytic domain of DNMT1 (Figure 2D).^32^, ^33^ Gene expression analysis by quantitative PCR showed that DNMT1 mRNA expression was not significantly downregulated in KURC3 tem/P4 compared with KURC3 Veh/P4 tumors (Figure 2E), and immunoblotting showed that DNMT1 protein expression was not decreased in KURC3 tem/P4 compared with KURC3 Veh/P4 tumors (Figure 2F). However, the DNA methyltransferase (DNMT) activity/inhibition assay showed that DNMT enzyme activity was significantly decreased in KURC3 tem/P4 compared with KURC3 veh/P4 tumors (Figure 2G). Taken together, these findings suggest that lower DNMT enzyme activity possibly caused by the heterogeneous *DNMT1* variant in the KURC3 PDX tumor was associated with the acquired phenotype of temsirolimus resistance.

### 3.5 Decreased DNMT enzyme activity with DNMT1 knockdown caused temsirolimus resistance in ccRCC cell line tumors

**FIGURE 3:**
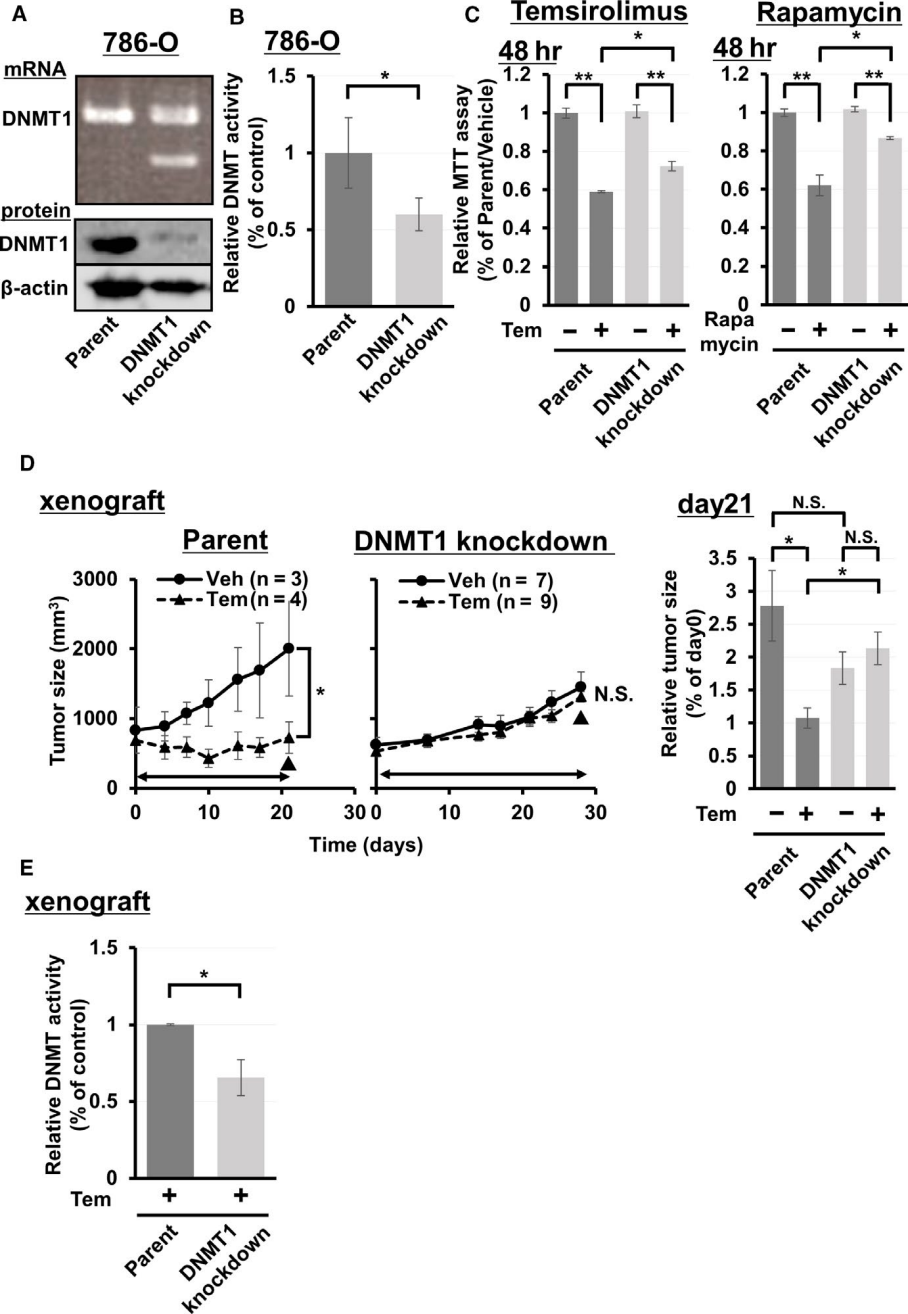
CRISPR/Cas9‐mediated DNMT1 knockdown leads to resistance to temsirolimus. (A) PCR of 786‐O or 786‐O subclones transfected with DNMT1 knockdown. Evaluation of DNMT1 protein expression in 786‐O or 786‐O subclones transfected with DNNT1 knockdown by immunoblotting. (B) Evaluation of DNMT activity in 786‐O or 786‐O subclones transfected with DNMT1 knockdown. All samples were prepared in triplicate and data are presented as the mean ± SE. Columns, mean; bar, SE. The difference in DNMT activity between parent and DNMT1 knockdown was statistically significant (*p < 0.05; Student's *t*‐test). (C) Evaluation of proliferation ability of 786‐O or 786‐O subclones transfected with DNMT1 knockdown in 48 h treated with vehicle or temsirolimus (left), and vehicle or rapamycin (right). Relative proliferation compared with parent cells treated with vehicle is indicated. All samples were prepared in triplicate and data are presented as the mean ± SE. Columns, mean; bar, SE. Statistical analysis was performed using Student's *t*‐test (*p < 0.05, **p < 0.01). (D) Sequential changes of subcutaneous xenograft tumors from 786‐O or 786‐O subclones transfected with DNMT1 knockdown treated with vehicle or temsirolimus (left). Relative tumor size on day 21 compared with tumor size on day 0 is indicated (right). Day 0 is the administration day. Each time point represents the mean ± SE of tumor volume in each group. Statistical analysis was performed using two‐way repeated ANOVA for sequential changes and Student's *t*‐test for relative tumor size (*p < 0.05, ns, not significant). Arrowed bars indicate the periods of temsirolimus administration. ▼ indicates the time point when tumors were resected. (E) Evaluation of DNMT activity in xenograft tumors of 786‐O or 786‐O subclones transfected with DNMT1 knockdown treated with temsirolimus. All samples were prepared in triplicate and data are presented as the mean ± SE. Columns, mean; bar, SE. The difference in DNMT activity between parent and DNMT1 knockdown was statistically significant (*p < 0.05; Student's *t*‐test)

Next, to clarify the effect of DNMT enzyme activity on temsirolimus sensitivity, temsirolimus‐sensitive 786‐O cells^34^ underwent DNMT1 deletion using CRISPR/Cas9. Because the heterozygous *DNMT1* variant was identified in KURC3 PDX tumors with temsirolimus resistance, we aimed to pick up 786‐O subclones heterozygous for DNMT1 knock‐out. PCR analysis showed that the expression of DNMT1 mRNA was downregulated heterozygously in CRISPR/Cas9‐mediated 786‐O subclones compared with parental cells (Figure 3A). Immunoblotting showed that DNMT1 protein expression was decreased in CRISPR/Cas9‐mediated 786‐O subclones compared with parental cells (Figure 3A). Additionally, the DNMT activity/inhibition assay revealed that DNMT enzyme activity of DNMT1 knockdown 786‐O cells was significantly decreased compared with parental cells (Figure 3B). The growth rate in DNMT1 knockdown cells was similar to that of parental cells without drug treatment in vitro (Figure 3C). With temsirolimus administration, growth in DNMT1 knockdown cells was suppressed but could be rescued compared with parental cells (Figure 3C). To evaluate the effect of other rapalogue, in vitro proliferation assay with rapamycin was also performed and showed similar results in cell growth with DNMT1 knockdown 786‐O cells (Figure 3C). Moreover, the growth in DNMT1 knockdown cells was suppressed with sunitinib administration, but not rescued, compared with parental cells (Figure S4). Notably, the tumor growth of xenografts from DNMT1 knockdown 786‐O cells was not suppressed by treatment with temsirolimus, while that of 786‐O xenografts was significantly inhibited by temsirolimus (Figure 3D). The DNMT activity/inhibition assay confirmed that the DNMT activity of DNMT1 knockdown 786‐O xenograft tumors was significantly decreased compared with that of 786‐O xenograft tumors (Figure 3E). These results suggest that DNMT1 heterozygous knockdown was associated with the suppression of DNMT enzyme activity which may underlie temsirolimus resistance in ccRCC cell line tumors.

### 3.6 Changes in methylation status and associated gene expression in temsirolimus‐resistant PDX tumors

**FIGURE 4:**
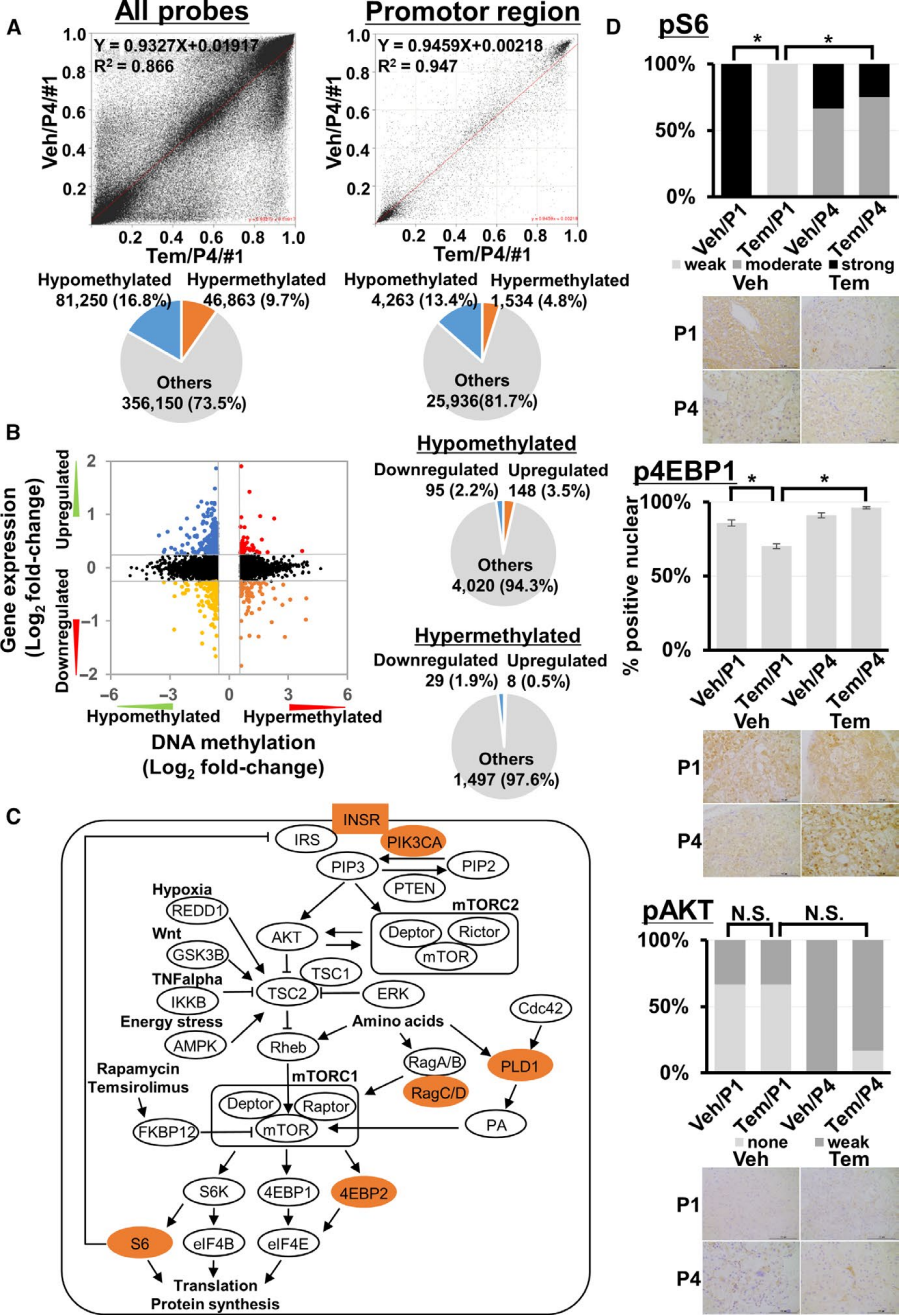
(A) Scatter plot of DNA methylation levels (Beta) of KURC3 Veh/P4/#1 and Tem/P4/#1 tumors in all probes (left) or promotor regions (right). Pie chart of the number and proportion of hypomethylated or hypermethylated probes used in each region. (B) Starburst plot for DNA methylation and microarray analyses in KURC3 PDX tumors. X‐axis is the log2 of the fold‐change (Tem/Veh) for methylation and y‐axis is the log2 of the fold‐change (Tem/Veh) for gene expression. Indicated points are genes that are hypomethylated and upregulated genes (blue); hypermethylated and downregulated genes (orange); hypermethylated and upregulated genes (red); or hypomethylated and downregulated genes (yellow). Pie chart of the number and proportion of downregulated or upregulated genes (Student's *t*‐test *p* < 0.05) in hypomethylated or hypermethylated probes. (C) Summary figure of the mTOR pathway. Hypomethylated and upregulated genes are marked. (D) Representative images and summary of immunohistochemical analysis of xenograft models. pS6, p4EBP1, and pAKT staining of KURC3 Veh/P1, Tem/P1, Veh/P4, and Tem/P4 tumors. Statistical analysis was performed using the Student's *t*‐test (*p < 0.05, ns, not significant). Scale bar, 100 µm. P1, passage 1; P4, passage 4; Tem, temsirolimus; Veh, vehicle

To explore whether the *DNMT1* variant and the suppression of DNMT enzyme activity led to changes in genome‐wide methylation profiling, we next performed methylation analysis. The number of hypomethylated probes was larger than that of hypermethylated ones in both all probes and promotor region probes (Figure 4A,B), suggesting that the global methylation status of temsirolimus‐resistant xenograft tumors could shift to become hypomethylated. The top 12 genes showing hypomethylation and hypermethylation in their promotor regions are listed in Table S4. To elucidate whether gene expression profiling was changed in temsirolimus‐resistant PDX tumors, microarray analysis for KURC1/P4 and KURC3/P4 tumors was performed. Genes whose expression was altered in KURC3/Tem/P4 tumors compared with KURC3/Veh/P4 tumors are listed in Table S5, although many were unchanged in expression between KURC1/Tem/P4 and KURC1/Veh/P4 tumors.

To evaluate the relationship between promotor methylation and gene expression profiling, we combined methylation and microarray analyses data. This showed that the number of upregulated genes with hypomethylated probes in promotor regions was larger than that of upregulated genes with hypermethylated probes (Figure 4B; Table S6). Table 3 lists genes that were both hypomethylated and upregulated in expression or vice versa in temsirolimus‐resistant KURC3 PDX tumors.

**TABLE 3:** Methylation status and mRNA changes after temsirolimus treatment in temsirolimus‐resistant (KURC3) or ‐sensitive (KURC1) xenograft tumors

Refseq	Gene	Gene description	KURC3 methylation fold change (Tem/Veh)	KURC3 mRNA fold change (Tem/Veh)	*p*‐value	KURC1 mRNA fold change (Tem/Veh)
			**DOWN**	**UP**		
NM_003615	*SLC4A7*	Solute carrier family 4, sodium bicarbonate cotransporter, member 7	0.23	1.9	0.013	0.99
NM_014746	*RNF144A*	Ring finger protein 144A	0.38	2.35	0.04	0.97
NM_025222	*WDR82*	WD repeat domain 82	0.39	1.78	0.004	0.83
NM_001112736	*FAM208A*	Family with sequence similarity 208, member A	0.41	1.99	0.005	0.86
NM_001165038	*GFRA2*	GDNF family receptor alpha 2	0.41	1.82	0.048	1.06
NR_024159	*DGCR9*	DiGeorge syndrome critical region gene 9	0.43	1.94	0.044	1.24
NM_001145464	*EXOG*	Endo/exonuclease (5′−3′), endonuclease G‐like	0.46	1.78	0.0007	0.82
NM_001111019	*NAV2*	Neuron navigator 2	0.46	2.31	0.022	1.16
NM_014903	*NAV3*	Neuron navigator 3	0.47	2.06	0.012	1.42
NM_001079872	*CUL4B*	Cullin 4B	0.48	1.96	0.002	0.95
NM_006317	*BASP1*	Brain abundant, membrane attached signal protein 1	0.49	2.13	0.047	1.48
			**UP**	**DOWN**		
NR_027822	*HLA‐L*	Major histocompatibility complex, class I, L (pseudogene)	5.05	0.67	0.001	0.55
NM_153028	*ZNF75A*	Zinc finger protein 75a	2.3	0.68	0.0007	0.85
NM_145905	*HMGA1*	High mobility group AT‐hook 1	2.19	0.55	0.001	1.82
NM_003202	*TCF7*	Transcription factor 7 (T‐cell specific, HMG‐box)	2.11	0.69	0.049	1.34
NM_001031849	*MASP1*	Mannan‐binding lectin serine peptidase 1 (C4/C2 activating component of Ra‐reactive factor)	2.03	0.64	0.023	0.93

Note

Methylation analysis for KURC3 Tem/P4/#1 and Veh/P4/#1 tumors (each *n* = 1). Microarray analysis for KURC3 Tem tumors (Tem/P4/#1, #2, #3, and #4: *n* = 4), KURC3 Veh tumors (Veh/P4/#1 and #2: *n* = 2), KURC1 Tem tumors (Tem/P4/#1 and #2: *n* = 2), and KURC1 Veh tumors (Veh/P4/#1: *n* = 1). Both hypomethylated (fold change <0.5) and upregulated (fold change >1.7) genes, or both hypermethylated (fold change >2.0) and downregulated (fold change <0.7) genes were listed, respectively.

Abbreviations: Tem, temsirolimus; Veh, vehicle.

### 3.7 Several molecules involved in the mTOR pathway showed altered methylation status and expression in temsirolimus‐resistant PDX tumors

We further evaluated methylation and gene expression profiling in mTOR pathway genes. In KURC3/Tem/P4 tumors, seven genes (*RPS6*, *INSR*, *PRKAA2*, *EIF4EBP2*, *PLD1*, *PIK3CA*, and *RRAGD*) could be both hypomethylated and upregulated, while these genes were unchanged in KURC1/Tem/P4 tumors (Table 4; Table S7). Candidate molecules associated with temsirolimus‐resistant PDX tumors in this study included those capable of reactivating the mTOR pathway in a manner that circumvented the mTORC1 inhibition mechanism by FKBP12 complex formation with rapalogues, suggesting that they may be involved in temsirolimus resistance (Figure 4C). Immunohistochemical analysis showed that pS6 protein expression was significantly suppressed in KURC3 Tem/P1 tumors compared with KURC3 Veh/P1 tumors, but that it was similar between KURC3 Tem/P4 and KURC3 Veh/P4 tumors. The proportion of tumor cells with nuclear phosphorylated eukaryotic translation initiation factor 4E‐binding protein 1 (p4EBP1)‐positive staining was decreased in KURC3 Tem/P1 tumors compared with KURC3 Veh/P1 tumors, but similar between KURC3 Tem/P4 and KURC3 Veh/P4 tumors. There was no difference in pAKT protein expression between these KURC3 groups (Figure 4D), and no difference in the protein expression of pS6, p4EBP1, or pAKT between each KURC1 group (data not shown).

**TABLE 4:** Methylation status and mRNA changes of mTOR signaling genes after temsirolimus treatment in temsirolimus‐resistant (KURC3) or ‐sensitive (KURC1) xenograft tumors

Refseq	Gene	Gene description	KURC3 methylation fold change (Tem/Veh)	KURC3 mRNA fold change (Tem/Veh)	*p*‐value	KURC1 mRNA fold change (Tem/Veh)
			DOWN	UP		
NM_001010	*RPS6*	Ribosomal protein S6	0.26	1.15	0.27	0.78
NM_000208	*INSR*	Insulin receptor	0.51	1.17	0.03	0.82
NM_006252	*PRKAA2*	Protein kinase, AMP‐activated, alpha 2 catalytic subunit	0.6	1.14	0.12	0.8
NM_004096	*EIF4EBP2*	Eukaryotic translation initiation factor 4E binding protein 2	0.69	1.02	0.83	0.79
NM_001130081	*PLD1*	Phospholipase D1, phosphatidylcholine‐specific	0.7	1.31	0.09	0.5
NM_006218	*PIK3CA*	Phosphatidylinositol‐4,5‐bisphosphate 3‐kinase, catalytic subunit alpha	0.72	1.04	0.52	0.82
NM_021244	*RRAGD*	Ras‐related GTP binding D	0.72	1.98	0.11	0.79

Note

Methylation analysis for KURC3 Tem/P4/#1 and Veh/P4/#1 tumors (each *n* = 1). Microarray analysis for KURC3 Tem tumors (Tem/P4/#1, #2, #3, and #4: *n* = 4), KURC3 Veh tumors (Veh/P4/#1 and #2: *n* = 2), KURC1 Tem tumors (Tem/P4/#1 and #2: *n* = 2), and KURC1 Veh tumors (Veh/P4/#1: *n* = 1). KURC3 hypomethylated (fold‐change <0.8), KURC3 upregulated (fold‐change >1.0), and KURC1 downregulated (fold‐change <1.0) genes in the mTOR pathway are listed.

Abbreviations: mTOR, mechanistic target of rapamycin; Tem, temsirolimus; Veh, vehicle.

## 4 DISCUSSION

The majority of patients with metastatic RCC are systemically treated with tyrosine kinase inhibitors, programmed death 1 checkpoint inhibitors, and mTOR inhibitors. Although the mTOR inhibitor temsirolimus is considered to be the standard treatment option for ccRCC patients with poor prognosis, most tumors eventually acquire resistance to the drug. Considering the rarity with which primary or metastatic tumors that have acquired resistance to temsirolimus are excised from ccRCC patients, it is difficult to obtain tissue to elucidate the genomic mechanisms underlying resistance. As an alternative, drug‐resistant models can be established using PDX models which preserve the histological and genomic profiling of primary tumors,^14^, ^15^ enabling their molecular biological changes to be observed. Indeed, mechanisms of drug resistance in breast and lung cancer were successfully analyzed using WES for PDX models.^35^, ^36^ However, although PDX models have been applied to evaluate drug resistance in ccRCC,^17^ no previous studies have used WES to investigate the genetic alterations associated with temsirolimus resistance.

Our PDX model enabled several ccRCC‐related gene alterations to be identified. In particular, KURC3 PDX tumors that finally acquired temsirolimus resistance were shown to harbor *BAP1* mutations. A previous report suggested that BAP1 prevents chromosome instability in breast cancer cells,^37^ indicating that *BAP1* mutations could lead to the occurrence of other de novo mutations in PDX tumors. In another report of metastatic RCC patients, *BAP1* mutations were associated with shorter progression‐free survival following treatment with everolimus.^38^ We speculate that KURC3 tumors with *BAP1* mutations acquired other mutations and resistance to mTORC1 inhibitors more readily than PDX tumors without *BAP1* mutations.

Several studies have proposed molecular mechanisms that mediate acquired resistance to mTORC1 inhibitors. WES for clinical anaplastic thyroid carcinoma revealed that mTOR mutations conferred resistance to mTOR inhibition,^39^ while certain mTOR mutations prevented binding of the FKBP12–rapamycin complex to mTORC1.^8^ Moreover, other report showed that using WES for pre‐treatment and post‐treatment tumor samples in six RCC cases, genetic alterations involving mTOR pathway were not newly acquired through mTOR inhibitor treatment.^40^ In human lung cancer cell lines, acquired resistance to PI3K/mTOR inhibition was documented after increased glycolysis associated with mitochondrial DNA mutations.^41^ Additionally, negative feedback loops, alternative pathway activation, and tumor heterogeneity were found to be related to mTOR resistance.^11^ Indeed, other study suggested that using ccRCC cell lines from PDX, mTOR inhibition‐induced alternative MEK activation.^19^ However, we did not identify the genetic alterations, suggested to be involved in mTOR resistance in those reports, in our resistant PDX models.

In the present study, comparing acquired resistant PDX tumors with sensitive ones using WES identified several genetic variations in temsirolimus‐resistant tumors, including those in *DNMT1*, *ANKRD12*, *CPD*, and *WDSUB1*. DNMT1 is a DNA methyltransferase with a key role in epigenetic regulation, and is responsible for maintaining the existing pattern of methylation during chromosome replication.^42^ The deletion of *Dnmt1* in a mouse model led to the global loss of DNA methylation and embryonic lethality,^43^ while even conditional loss of *Dnmt1* in the developing mouse brain resulted in DNA hypomethylation and postnatal lethality.^44^ Human disease associated with DNMT1 is often caused by heterozygous mutations that result in modest methylation changes.^42^, ^45^ Considering that a DNMT1 deficiency was reported to cause increased genome instability in the APCMin/+ intestinal epithelia,^46^ early passage *DNMT1* mutations and the functional loss of DNMT1 could lead to the accumulation of other mutations in cancer cells. Indeed, in TCGA cohort of ccRCC patients, DNA methyltransferase (*DNMT1*, *DNMT3A*, and *DNMT3B*) alterations were detected in ～8% of entire cohort, and the mutation burden in tumors with them are significantly larger than those in tumors without (Figure S5).^28^, ^29^, ^47^ The reduction of DNMT1 was previously shown to be correlated with induction of the epithelial–mesenchymal transition (EMT) and cancer stem cell (CSC) phenotype in prostate cancer cells.^48^ In view of the observations that mesenchymal non‐small cell lung carcinoma cancers are likely to be resistant to epidermal growth factor receptor and PI3 K/AKT inhibitors,^49^ and that the EMT/CSC phenotype could cause mTORC1 inhibitor resistance,^50^ it is possible that DNMT1 haploinsufficiency in cells is associated with resistance to mTOR pathway inhibition. In our KURC3 PDX tumors, the *DNMT1* mutation was not identified initially, and it is suggested that this mutation would not be related to carcinogenesis or early progression of the KURC3 tumor. In this study, database analysis using the Human Protein Atlas in TCGA cohort of ccRCC patients indicated that overall survival in individuals with low DNMT1 expression was similar to that in patients with high DNMT1 expression (Figure S6). Moreover, DNMT1 mRNA expression was similar between temsirolimus‐resistant PDX tumors and temsirolimus‐sensitive tumors (Table S8). In passage 1 cohorts of KURC3 PDX tumors, the *DNMT1* mutation was identified at a mutation frequency of about 50%, which is almost the same as following passage 2, 3, and 4 cohorts. If they consist of cells heterogeneously with wild‐type and homozygous mutation, it could be assumed the mutation frequency could change in each passage depending on their cell ratio. So, *DNMT1* mutation in the resistant tumor cells seemed to be homogeneous heterozygous mutation. In present study, *DNMT1* mutations and loss of DNMT enzyme activity were observed in resistant KURC3 PDX tumors, and we showed that, in vitro, loss of DNMT enzyme activity leads to the phenotype of temsirolimus‐resistance in certain RCC cells. Collectively, it was suggested that the *DNMT1* mutation followed by reduced enzyme activity could be partially contributed to temsirolimus resistance in these models, while further study is necessary to elucidate the functional evidence to link the *DNMT* mutation with temsirolimus resistance.

The role of ankyrin repeat domain 12 (ANKRD12) and the functional consequence of the *ANKRD12* variation observed in the present study remain unclear. A previous report showed that ANKRD12 was downregulated in colorectal cancer and that lower expression correlated with poor survival and liver metastasis in colorectal cancer patients.^51^ Database analysis in our study confirmed this, by revealing shorter overall survival in patients with low ANKRD12 expression than in those with high expression (Figure S6). We also found that ANKRD12 mRNA expression was downregulated in temsirolimus‐resistant PDX tumors (Table S8). Carboxypeptidase D (CPD) is a membrane‐bound metalloproteinase that cleaves arginine, which is transported into cells for conversion into nitric oxide. In breast cancer cells and prostate cancer cells, CPD knockdown suppressed nitric oxide levels and cell viability and increased apoptosis.^52^ However, the function of CPD in ccRCC remains unclear. The WD repeat, sterile alpha motif and U‐box domain containing 1 gene (WDSUB1) encodes one of seven U‐box ubiquitin ligases in humans,^53^ whose function is also unclear. In ccRCC patients, overall survival was found to be shorter in those with low WDSUB1 expression compared with those with high expression (Figure S6), while WDSUB1 mRNA expression was similar in temsirolimus‐resistant PDX tumors to control tumors (Table S8). Further studies are needed to determine how functional changes in these genes affect developing resistance to mTOR inhibitors.

Microarray analysis showed that the expression of several genes was changed in temsirolimus‐resistant PDX tumors (Table S5). Among them, long noncoding (lnc) RNA‐H19 had the greatest increase in expression. It has been suggested to be associated with the mTOR pathway activity via its mature product, miR‐675. In retinoblastoma cells, lncRNA‐H19 knockdown suppressed cell viability, migration, and invasion together with inhibition of PI3K/AKT/mTOR pathways.^54^ lncRNA‐H19 also inhibited cell growth by blocking the function of mTORC1 in pituitary tumors.^55^ Although further investigation is necessary, lncRNA‐H19 upregulation may mediate resistance to temsirolimus in our PDX tumors by increasing mTOR pathway activity. Combined evaluation with microarray and methylation analyses identified several genes whose promotor methylation was suppressed, leading to upregulation of expression, in temsirolimus‐resistant PDX tumors (Table 3). Cullin 4B (CUL4B) is a scaffold of the Cullin 4B‐Ring E3 ligase complex that plays a key role in proteolysis and is upregulated in many malignancies.^56^ CUL4B induces EMT via the Wnt/beta‐catenin signaling pathway in pancreatic cancer cells.^57^ Intriguingly, silencing CUL4B was shown to suppress mTOR‐mediated S6K1 phosphorylation.^58^

Integrated analysis of the mTOR pathway suggested that several genes including *PLD1* (encoding phospholipase D1), *RRAGD* (encoding Ras‐related GTP binding D), *RPS6* (encoding ribosomal protein S6), *INSR* (encoding insulin receptor), and *PI3KCA* (encoding phosphatidylinositol 3‐kinase), which are both upregulated and hypomethylated, are candidates for temsirolimus resistance. PLD1 generates phosphatidic acid which interacts directly with mTOR and is required for the stability of mTORC1.^59^ PLD activity is elevated in many human cancers,^60^ and this was previously shown to directly activate mTORC1 under temsirolimus administration.^61^ Nutrients including amino acids activate mTORC1 by driving its recruitment to the lysosomal membrane via Rag GTPases, which form heterodimers consisting of RagA or RagB tightly bound to RagC or RagD.^62^ WES previously revealed recurrent mTORC1‐activating mutations in *RRAGC*, which encodes RagC, in follicular lymphoma; the mutants increased raptor binding while rendering mTORC1 signaling resistant to amino acid deprivation.^63^ Earlier reports showed that elevated RagD expression was associated with temsirolimus resistance.^64^ Because mTORC1 and S6K mediate potent negative feedback loops through insulin receptor, their suppression leads to compensatory activation of upstream signaling, including PI3K and Akt, which potentially opposes the effects of the inhibitors and leads to drug resistance.^65^ Taken together, the elevated expression of these mTOR pathway genes in our temsirolimus‐resistant tumors could cause the cancelation of negative feedback loops and/or the reactivation of mTORC1 by another mechanism that escapes inhibition by rapalogues.

The present study has a number of limitations in. First, we only analyzed two cohorts of temsirolimus‐resistant or ‐sensitive PDX tumors. Even though we demonstrated the feasibility of using these models to explore the underlying genetic mechanisms of resistance acquisition, our proposed system requires further validation in ccRCC patients. Second, genetic analyses with PDX models have a number of challenges including the time and cost required to establish them, species differences in tumor microenvironments,^15^ and DNA contamination from mice. The difference in pharmacokinetics in human and several mouse strains should be considered especially in the drug trial with PDX or cell line xenograft models for studying resistance mechanisms. Recently several procedures have successfully removed mouse genome information from sequencing data,^35^, ^36^ while another study analyzed drug resistance in mammary tumors using tumor organoids^66^ which may help to overcome the limitation.

## 5 CONCLUSION

In the present study, we established a PDX model of acquired resistance to the mTOR inhibitor temsirolimus, and showed the feasibility of using this model to explore the underlying genetic mechanisms of resistance acquisition. The genetic alterations including *DNMT1* mutations, and changes in methylation status or gene expression in cancer cells could be one of the potential mechanisms of developing resistance to temsirolimus. Such mechanisms could eventually be targeted to overcome resistance in cohorts of ccRCC patients.

## CONFLICT OF INTEREST

The authors declare that they have no conflict of interest.

## Supporting information

Supplementary Material

## Data Availability

The datasets used and analyzed during the current study are available from the corresponding author on reasonable request.
