# Supplementary material for: Functional and genomic characterization of patient‐derived xenograft model to study the adaptation to mTORC1 inhibitor in clear cell renal cell carcinoma
Source: Cancer Med. 2020 Oct 27;10(1):119–34. doi: 10.1002/cam4.3578 (PMC7826464; doi:10.1002/cam4.3578)
Supplement: Supplementary file 1 — Supplementary Material [file CAM4-10-119-s001.docx]

**SUPPLEMENTARY METHODS**

1. **DNA and RNA extraction**

Total RNA and DNA from KURC1 and KURC3 tumors treated with vehicle or temsirolimus were extracted using the RNeasy Mini kit and QIAamp DNA mini kit (Qiagen, Hilden, Germany), respectively, according to the manufacturer’s instructions.

1. **PDX tumor mutation analysis**

To evaluate ccRCC-related gene alterations in PDX models, KURC1 and KURC3 passage 4 vehicle data were filtered using the previously reported gene list: *VHL*, *PBRM1*, *SETD2*, *KDM5C*, *PTEN*, *BAP1*, *MTOR*, *TP53*, *PIK3CA*, *MSR1*, *TXNIP*, *TCEB1*, *NFE2L2*, *BTNL3*, *SLITRK6*, *RHEB*, *ARID1A*, *NPNT*, *CCNB2*, *ZNF800*, *SLC27A6*, *COL6A6*, *SPRED1*, *FBN2*, *STAG2*, *SECISBP2L*, *TFDP2*, *HMCN1*, *ATM*, and *MAGEC1*.[^1^](#_ENREF_1)

1. **PCR and Sanger sequencing**

Primers targeting the genomic region were designed according to previous studies or using primer BLAST. The primer sequences were as follows: *WDSUB1* 5′-TTGGCTTCCTAGGGTCATCA-3′ and 5′-CTATGCTGTCCACTGCTGCT-3′; *HTR1A* 5′-TGTTCACGTAGTCGATGGGG-3′ and 5′-ACAGCGACAGACAGACGTTC-3′; *FTMT* 5′-CTCAGTTTCCCCACTTCCAAGG-3′ and 5′-CAGCTTCTCCGCGTGCTCG-3′; *QSER1* 5′-TGCAGAACTAACTGGTCAGG-3′ and 5′-AGCACCAAAGGTGAAGTGGT-3′; *CPD* 5′-CTACCAGGCACTGTGGGAAG-3′ and 5′-GGCACTAGCTTGAGGCATGA-3′; *ANKRD12* 5′-TGGGTATGAGTGCCATTGAGG-3′ and 5′-TCGGGTATCTTTTGATGCTGGT-3′; *DNMT1* 5′-CCAGGTTGTCCTCCATCTG-3′ and 5′-ACTCAATCCTCACAGCAGCC-3′. PCR and Sanger sequencing were performed as previously described.[^2^](#_ENREF_2) Cycling conditions were as follows: 32 cycles at 95 °C for 30 s, 60°C for 30 s, and 72 °C for 40 s.

1. **Quantitative real-time PCR**

cDNA synthesis and quantitative PCR for DNMT1 and GAPDH were performed as previously described.[^3^](#_ENREF_3) Primer sequences were as follows: DNMT1: 5′-GAGCCACAGATGCTGACAAA-3′ and 5′-TGCCATTAACACCACCTTCA-3′; GAPDH: 5′-TCGGAGTCAACGGATTTGGT-3′ and 5′-TGAAGGGGTCATTGATGGCA-3′.

1. **Protein extraction and immunoblot analysis**

Whole cell proteins were isolated from snap-frozen specimens or cultured cells and analyzed by immunoblotting as previously described.[^3^](#_ENREF_3) Antibodies were purchased commercially as follows: anti-DNMT1 (ab13537) and human β-actin (Abcam, Cambridge, MA).

1. **Cell culture**

The 786-O cell line was purchased from the American Type Culture Collection (Rockville, MD). Cells were cultured in DMEM (Invitrogen, Carlsbad, CA) containing 10% fetal bovine serum (FBS) supplemented with 1% penicillin/streptomycin.

1. **Cell proliferation assays**

Cells were seeded in triplicate at 1000 cells/100 µl in DMEM with 10% FBS in each well of 96-well plates and cultured for 72 h. Next, 10 µl Cell Proliferation Reagent Cell Counting Kit-8 (Dojindo Laboratories, Kumamoto, Japan) was added to each well and the cells were incubated for another 2 h. Absorbance was detected at 450 and 650 nm with a 96-well spectrophotometric plate reader. Temsirolimus, rapamycin, or vehicle was added to the medium at a final concentration of 100 nM. Sunitinib or vehicle was added to the medium at a final concentration of 4 µM.

1. **mTOR pathway gene analysis**

mTOR pathway-related gene alterations were reviewed using the gene list of RT² Profiler™ PCR Array Human mTOR Signaling (Qiagen). KURC3 hypomethylated (fold-change < 0.8), KURC3 upregulated (fold-change > 1.0), and KURC1 downregulated (fold-change < 1.0) genes were filtered.

1. **Immunohistochemistry**

Immunohistochemical analysis was performed on formalin-fixed, paraffin-embedded clinical samples or xenograft tissues as previously described.[^3^](#_ENREF_3) Antibodies were purchased commercially as follows: human phospho-4E-BP1 (Thr37/46) (236B4), human phospho-S6 Ribosomal Protein (Ser240/244) (D68F8), human phospho-Akt (Ser473) (all from Cell Signaling Technology, Beverly, MA).

**References**

1. Comprehensive molecular characterization of clear cell renal cell carcinoma. *Nature.* 2013;499(7456):43-49.

2. Sakamoto H, Yamasaki T, Sumiyoshi T, et al. A family case with germline TSC1 and mtDNA mutations developing bilateral eosinophilic chromophobe renal cell carcinomas without other typical phenotype of tuberous sclerosis. *Journal of clinical pathology.* 2018.

3. Yamasaki T, Kamba T, Kanno T, et al. Tumor microvasculature with endothelial fenestrations in VHL null clear cell renal cell carcinomas as a potent target of anti-angiogenic therapy. *Cancer science.* 2012;103(11):2027-2037.

**
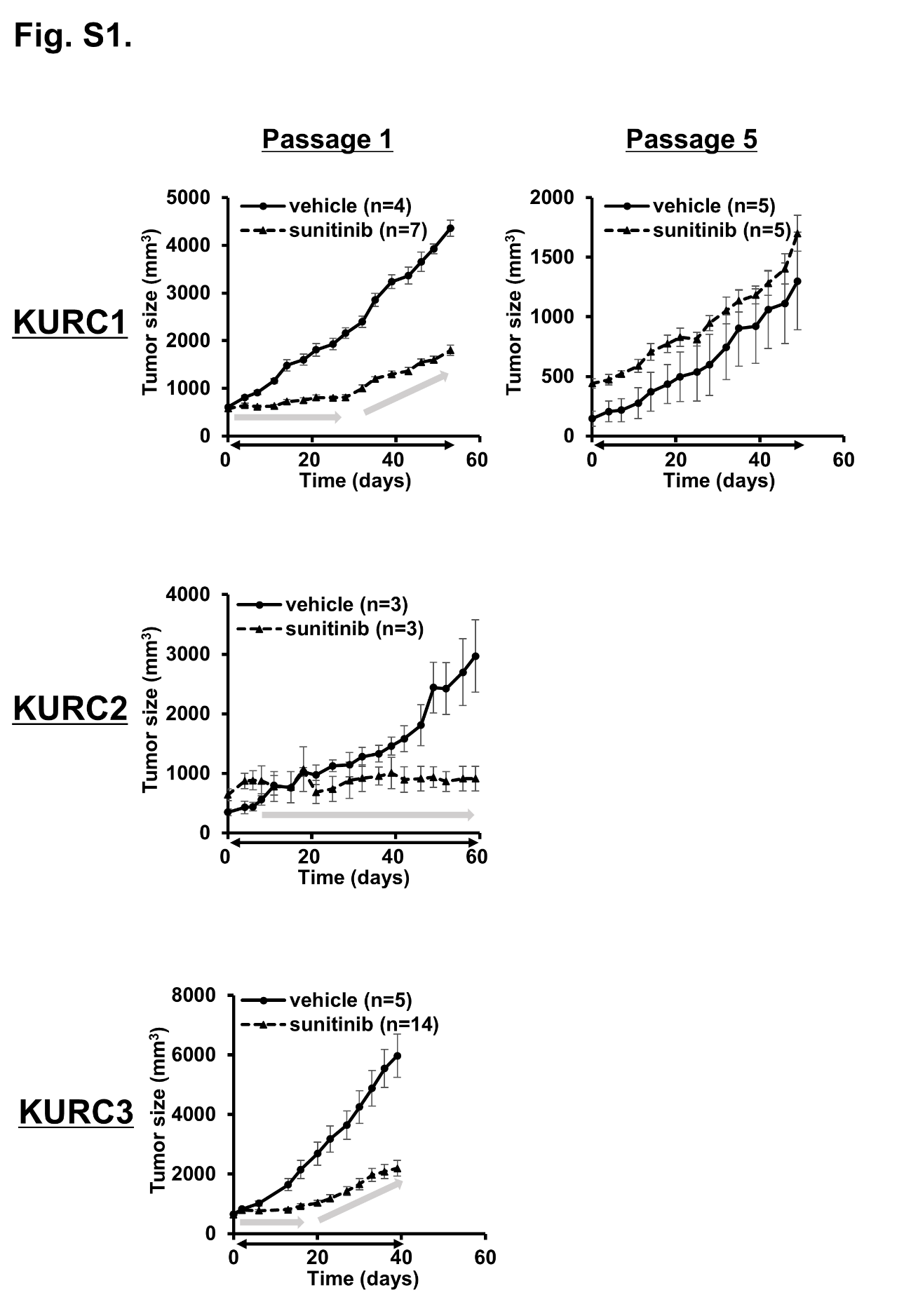
**

**Fig. S1.** Sequential changes of KURC1 (passage1 and 5), KURC2 (passage1), or KURC3 (passage1) xenograft tumors treated with vehicle or sunitinib. Each time point represents the mean ± SE of tumor volume in each group. Day 0 is the administration day. Black arrowed bars indicate the periods of sunitinib administration. Grey arrows indicate the trend of tumor growth


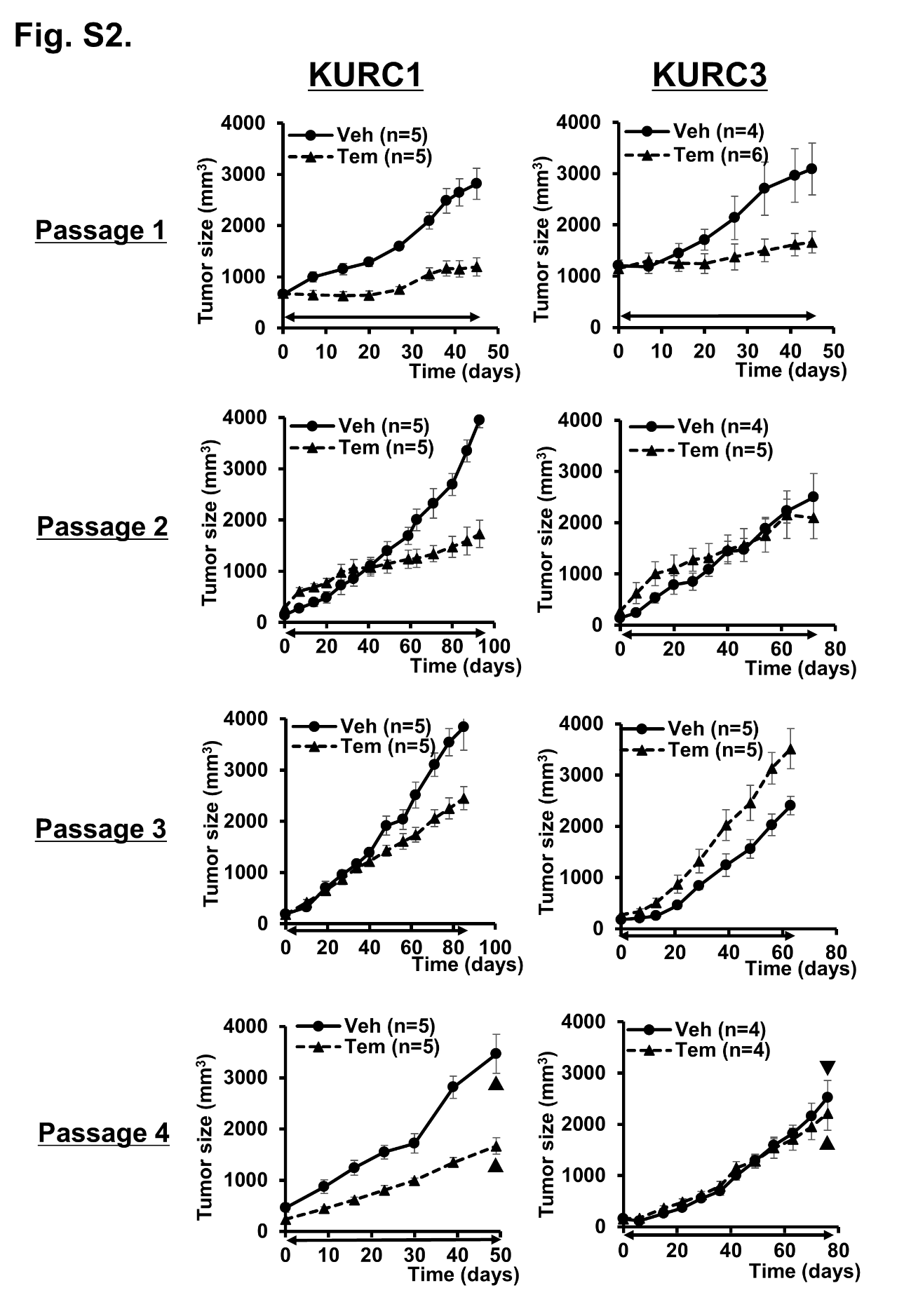


**Fig. S2.** Sequential changes of KURC1 or KURC3 xenograft tumors (passage 1, 2, 3, and 4 treated with vehicle or temsirolimus). KURC1 remained sensitive and KURC3 developed resistance to temsirolimus. Each time point represents the mean ± SE of tumor volume in each group. Day 0 is the administration day. Statistical analysis was performed using two-way repeated ANOVA (**P* < 0.05). Arrowed bars indicate the periods of temsirolimus administration


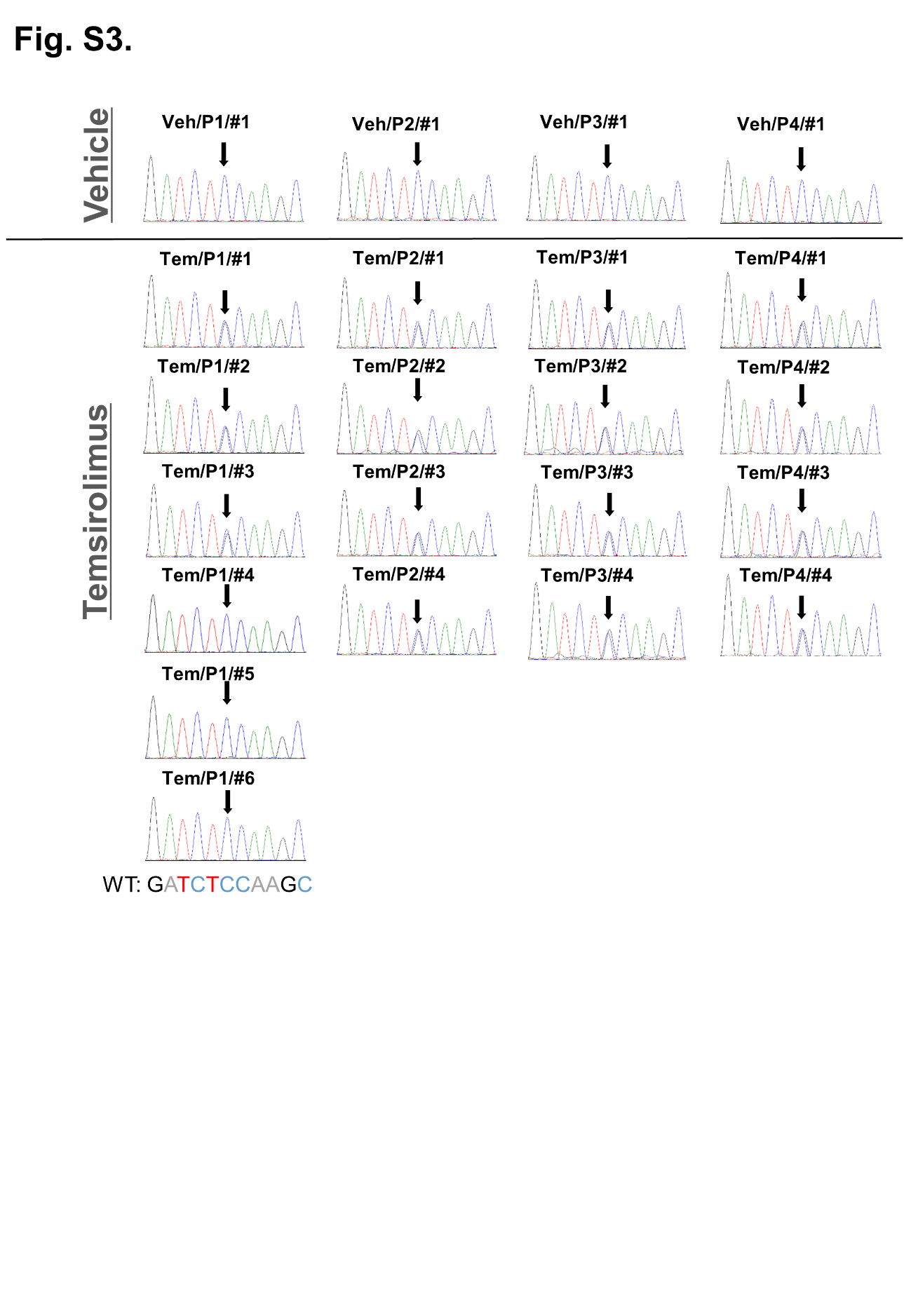


**Fig. S3.** Sanger sequencing shows heterozygous *DNMT1* missense mutation in KURC3 Tem/P2, Tem/P3, and Tem/P4 tumors. The same heterozygous mutation was identified in KURC3 Tem/P1#1, #2, #3, but not in Tem/P1/#4, #5, #6 or each vehicle tumor. P1, passage 1; veh, vehicle; tem, temsirolimus


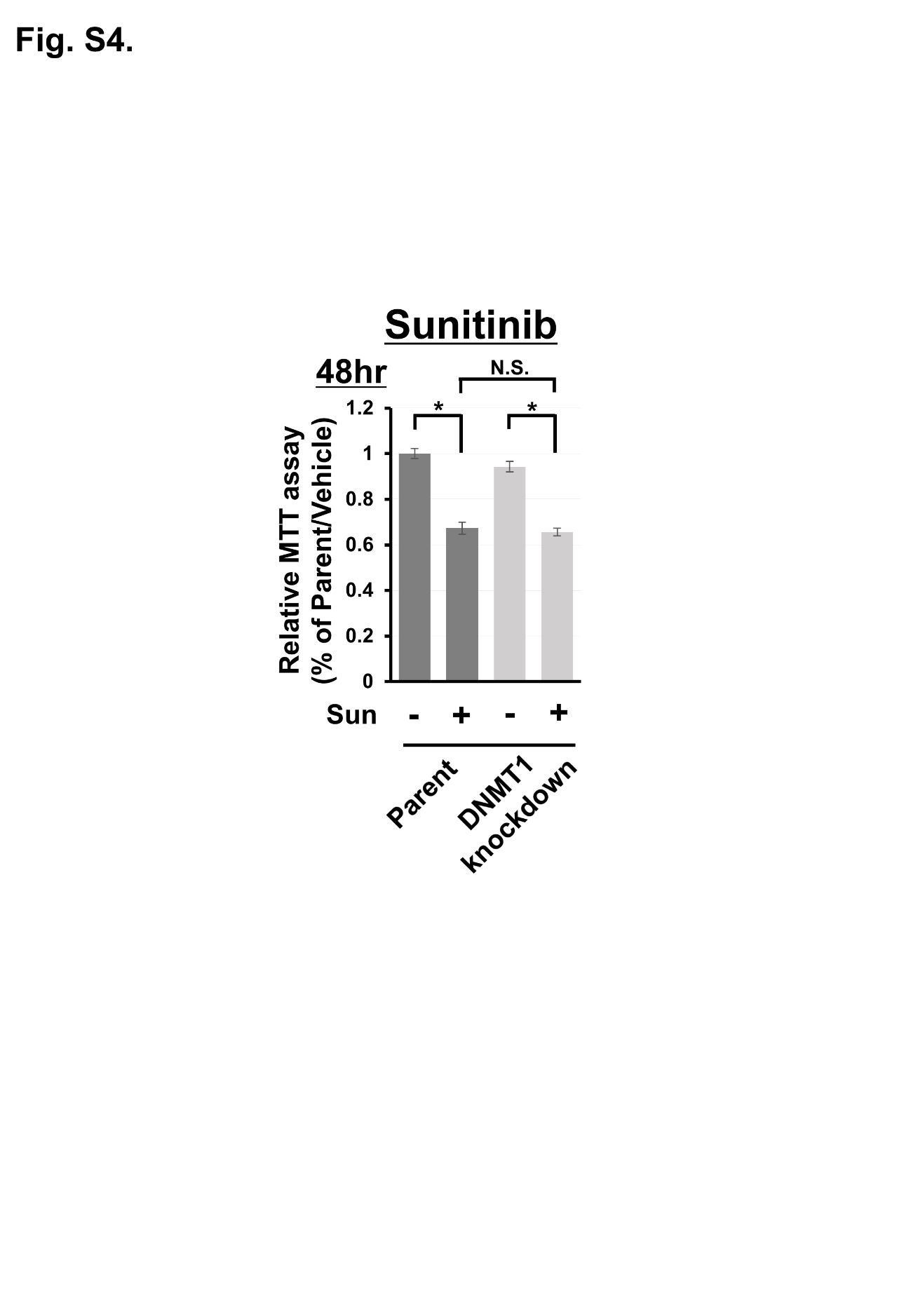


**Fig. S4.** Evaluation of proliferation ability of 786-O or 786-O subclones transfected with DNMT1 knockdown in 48 h treated with vehicle or sunitinib. Relative proliferation compared with parent cells treated with vehicle is indicated. All samples were prepared in triplicate and data are presented as the mean ± SE. Columns, mean; bar, SE. Statistical analysis was performed using Student’s t-test (*P < 0.01, N.S.: not significant).


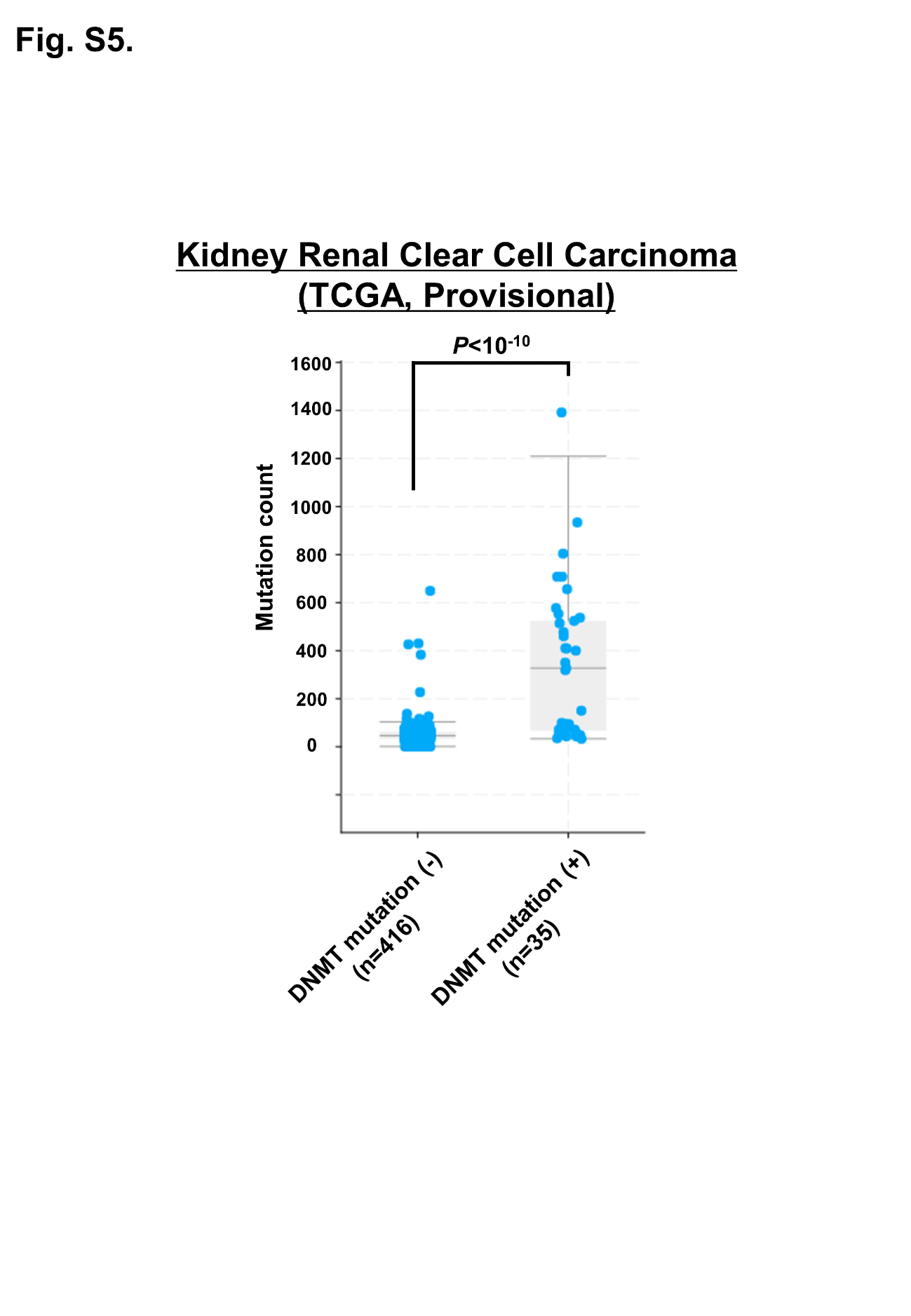


**Fig. S5.** Beeswarm box-plot: Mutation counts in ccRCC patients with DNMT mutation were significantly larger than those in patients without DNMT mutation. Image available from cBioPortal for cancer genomics. TCGA, The Cancer Genome Atlas; DNMT, DNA methyltransferase

**
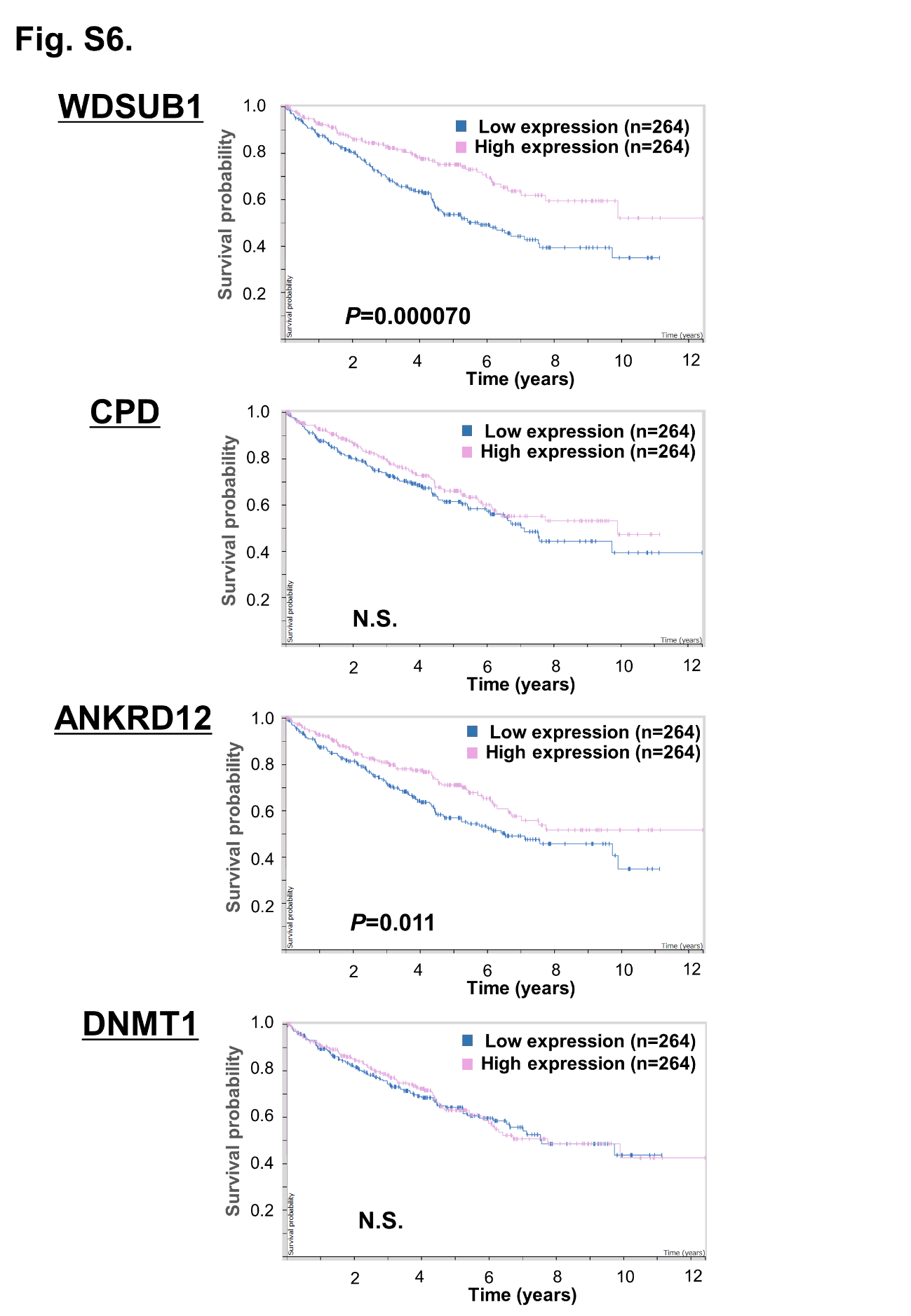
**

**Fig. S6.** Image available from v18.1.proteinatlas.org. Low protein expression of ANKRD12 or WDSUB1 in ccRCC patients was associated with shorter overall survival. Expression of DNMT1 or CPD was not associated with survival. N.S., not significant

| **Table S1.** The number of PDX tumors in each passage or for WES, methylation, and microarray analyses | | | | | | | |
| --- | --- | --- | --- | --- | --- | --- | --- |
|  | P1 | P2 | P3 | P4 | P4 | | |
|  |  |  |  |  | WES | methylation | microarray |
| KURC1 Veh | 5 | 5 | 5 | 5 | 1 | 0 | 1 |
| KURC1 Tem | 5 | 5 | 5 | 5 | 2 | 0 | 2 |
| KURC3 Veh | 4 | 4 | 5 | 4 | 1 | 1 | 2 |
| KURC3 Tem | 6 | 5 | 5 | 4 | 2 | 1 | 4 |
| Veh, vehicle; Tem, temsirolimus; P1, passage 1; WES, whole-exome sequencing | | | | | | | |

| **Table S2.** The proportion of reads mapped for human or mouse genomes | | | | |
| --- | --- | --- | --- | --- |
| Sample name | Mapped reads | % of human  mapped reads | % of mouse  mapped reads | coverage |
| KURC1 |  |  |  |  |
| KURC1 primary tumor | 85,365,274 | 99.88 | 0.09 | 52.0 |
| KURC1 Veh/P4/#1 | 44,923,390 | 83.96 | 16.03 | 47.6 |
| KURC1 Tem/P4/#1 | 46,272,391 | 65.09 | 34.89 | 38.4 |
| KURC1 Tem/P4/#2 | 45,350,597 | 66.94 | 33.04 | 38.3 |
| KURC3 |  |  |  |  |
| KURC3 Veh/P4/#1 | 47,628,646 | 63.77 | 35.56 | 40.7 |
| KURC3 Tem/P4/#1 | 43,900,658 | 70.11 | 29.59 | 43.6 |
| KURC3 Tem/P4/#2 | 48,468,352 | 81.92 | 17.22 | 37.1 |
| Veh, vehicle; Tem, temsirolimus; P4, passage 4 | | |  |  |

| **Table S3.** The variants identified in KURC1 Tem/P4/#1, #2 tumors. | | | | | | | | |
| --- | --- | --- | --- | --- | --- | --- | --- | --- |
| Gene | Chr | Start Position | Ensemble | Allele Change | mutation type | Allele Frequency (Tem) | Allele Frequency (Veh) | Amino Acid  Change |
| P4/tem/#1 |  |  |  |  |  |  |  |  |
| *KAZN* | 1 | 14925620 | '00000376030 | c.128_129insATG | inframe_insertion | 0.6 | 0.237 | p.Gly43dup |
| *STT3B* | 3 | 31574393 | '00000295770 | c.-82_-77dupTCCTCC | 5' UTR | 0.6 | 0.214 | . |
| ***MAGI1*** | **3** | **65583531** | **'00000460329** | **c.29A>G** | **missense** | **0.394** | **0** | **p.Gln10Arg** |
| *GFPT2* | 5 | 179780274 | '00000253778 | c.-89_-88insGGAGCCCACGGA | 5' UTR | 0.588 | 0.143 | . |
| ***POU6F2*** | **7** | **39125405** | **'00000559001** | **c.-61A>T** | **5' UTR** | **0.596** | **0.183** | **.** |
| *POU6F2* | 7 | 39125440 | '00000559001 | c.-26A>G | 5' UTR | 0.584 | 0.19 | . |
| *POU6F2* | 7 | 39125447 | '00000559001 | c.-19T>C | 5' UTR | 0.576 | 0.183 | . |
| *POU6F2* | 7 | 39125449 | '00000559001 | c.-17G>C | 5' UTR | 0.574 | 0.185 | . |
| ***STEAP4*** | **7** | **87912204** | **'00000380079** | **c.736A>G** | **missense** | **0.5** | **0** | **p.Ile246Val** |
| ***ZHX2*** | **8** | **123965331** | **'00000314393** | **c.1581C>G** | **missense** | **0.474** | **0** | **p.Asp527Glu** |
| ***TOLLIP*** | **11** | **1304706** | **'00000530506** | **n.*37delC** | **3' UTR** | **0.462** | **0** | **.** |
| ***FAM96A*** | **15** | **64386061** | **'00000300030** | **c.-94G>C** | **5' UTR** | **0.55** | **0** | **.** |
| *WIZ* | 19 | 15549715 | '00000389282 | c.1946C>T | missense | 0.35 | 0 | p.Ala649Val |
| *NUMBL* | 19 | 41173892 | '00000252891 | c.1294_1299delCAGCAA | inframe_deletion | 0.545 | 0.167 | p.Gln432_Gln433del |
| ***CARD10*** | **22** | **37888762** | **'00000251973** | **c.2524G>C** | **missense** | **0.519** | **0** | **p.Val842Leu** |
| ***AKAP17A*** | **X** | **1712773** | **'00000313871** | **c.418A>G** | **missense** | **0.368** | **0** | **p.Thr140Ala** |
| *AKAP17A* | X | 1712953 | '00000313871 | c.598A>G | missense | 0.353 | 0 | p.Thr200Ala |
| P4/tem/#2 |  |  |  |  |  |  |  |  |
| *EFCAB7* | 1 | 63997594 | '00000371088 | c.291G>C | missense | 0.692 | 0.22 | p.Arg97Ser |
| *NHEJ1* | 2 | 220022973 | '00000409720 | c.112G>A | missense | 0.647 | 0.208 | p.Val38Ile |
| ***MAGI1*** | **3** | **65583531** | **'00000460329** | **c.29A>G** | **missense** | **0.352** | **0** | **p.Gln10Arg** |
| *POU6F2* | 7 | 39125372 | '00000559001 | c.-94G>C | 5' UTR | 0.522 | 0.071 | . |
| ***POU6F2*** | **7** | **39125405** | **'00000559001** | **c.-61A>T** | **5' UTR** | **0.577** | **0.183** | **.** |
| ***STEAP4*** | **7** | **87912204** | **'00000380079** | **c.736A>G** | **missense** | **0.58** | **0** | **p.Ile246Val** |
| ***ZHX2*** | **8** | **123965331** | **'00000314393** | **c.1581C>G** | **missense** | **0.39** | **0** | **p.Asp527Glu** |
| ***TOLLIP*** | **11** | **1304706** | **'00000530506** | **n.*37delC** | **3' UTR** | **0.421** | **0** | **.** |
| ***FAM96A*** | **15** | **64386061** | **'00000300030** | **c.-94G>C** | **5' UTR** | **0.52** | **0** | **.** |
| *NEFH* | 22 | 29885598 | '00000310624 | c.1973_1978delAAGAGG | inframe_deletion | 0.464 | 0 | p.Glu658_Glu659del |
| ***CARD10*** | **22** | **37888762** | **'00000251973** | **c.2524G>C** | **missense** | **0.529** | **0** | **p.Val842Leu** |
| ***AKAP17A*** | **X** | **1712773** | **'00000313871** | **c.418A>G** | **missense** | **0.588** | **0** | **p.Thr140Ala** |
| *MT-ND5* | MT | 12463 | '00000361567 | c.127A>C | missense | 0.353 | 0 | p.Thr43Pro |
| Tem, temsirolimus; P4, passage4; Chr, chromosome; Veh, vehicle; N/A, not applicable  The common gene alterations between two tumors (Tem/P4/#1 and #2) were shown by bold letters. | | | | | | | | |

| **Table S4.** Methylation status changes after temsirolimus treatment in temsirolimus-resistant xenograft tumors (KURC3) | | | |
| --- | --- | --- | --- |
| Refseq | Gene | Gene description | KURC3 methylation  fold-change (Tem/ Veh) |
|  |  |  | DOWN |
| NM_033051 | *SLC46A2* | solute carrier family 46, member 2 | 0.03 |
| NM_001012994 | *SNX30* | sorting nexin family member 30 | 0.03 |
| NM_020783 | *SYT4* | synaptotagmin IV | 0.04 |
| NM_001040272 | *ADAMTSL1* | ADAMTS-like 1 | 0.05 |
| NM_001633 | *AMBP* | alpha-1-microglobulin/bikunin precursor | 0.06 |
| NM_032048 | *EMILIN2* | elastin microfibril interfacer 2 | 0.06 |
| NM_001142530 | *BHLHB9* | basic helix-loop-helix domain containing, class B, 9 | 0.06 |
| NR_003204 | *SNORD114-11* | small nucleolar RNA, C/D box 114-11 | 0.06 |
| NM_001099219 | *KRTAP19-8* | keratin associated protein 19-8 | 0.06 |
| NM_002548 | *OR1D2* | olfactory receptor, family 1, subfamily D, member 2 | 0.06 |
| NM_001042583 | *CD1E* | CD1e molecule | 0.07 |
| NM_031426 | *AIF1L* | allograft inflammatory factor 1-like | 0.07 |
|  |  |  | UP |
| NR_003193 | *SNORD114-1* | small nucleolar RNA, C/D box 114-1 | 3.85E+07 |
| NR_023316 | *ACSF3* | acyl-CoA synthetase family member 3 | 16.6 |
| NM_001725 | *BPI* | bactericidal/permeability-increasing protein | 15.8 |
| NM_001004712 | *OR4K14* | olfactory receptor, family 4, subfamily K, member 14 | 15.5 |
| NM_006417 | *IFI44* | interferon-induced protein 44 | 15.2 |
| NM_001085479 | *IQCF3* | IQ motif containing F3 | 15.1 |
| NM_002632 | *PGF* | placental growth factor | 14.9 |
| NR_003220 | *SNORD114-27* | small nucleolar RNA, C/D box 114-27 | 14.1 |
| NM_024847 | *TMC7* | transmembrane channel-like 7 | 13.6 |
| NM_001129820 | *SLFN14* | schlafen family member 14 | 13.6 |
| NM_001004715 | *OR4K17* | olfactory receptor, family 4, subfamily K, member 17 | 13.2 |
| NM_030766 | *BCL2L14* | BCL2-like 14 (apoptosis facilitator) | 13.1 |
| Tem, temsirolimus; Veh, vehicle | | |  |
| Methylation analysis for KURC3 Tem/P4/#1 and Veh/P4/#1 tumors (each n=1). | | | |

| **Table S5.** mRNA changes after temsirolimus treatment in temsirolimus-resistant PDX tumors (KURC3) compared with temsirolimus-sensitive xenograft tumors (KURC1) | | | | | |
| --- | --- | --- | --- | --- | --- |
| Refseq | Gene | Gene description | KURC3 | | KURC1 |
|  |  |  | mRNA fold-change (Tem / Veh) | *P*-value | mRNA fold-change (Tem / Veh) |
|  |  |  | UP |  |  |
| NR_002196 | *H19\|MIR675* | H19, imprinted maternally expressed transcript (non-protein coding) | 14.60 | 0.029 | 0.98 |
| NM_005711 | *EDIL3* | EGF-like repeats and discoidin I-like domains 3 | 6.87 | 0.002 | 0.66 |
| NM_001080393 | *GXYLT2* | glucoside xylosyltransferase 2 | 5.60 | 0.017 | 1.19 |
| NM_005160 | *ADRBK2* | adrenergic, beta, receptor kinase 2 | 5.23 | 0.010 | 0.97 |
| NM_020707 | *HHATL* | hedgehog acyltransferase-like | 4.27 | 0.022 | 1.08 |
| NM_018153 | *ANTXR1* | anthrax toxin receptor 1 | 4.10 | 0.001 | 1.79 |
| NM_001113226 | *NTNG1* | netrin G1 | 3.64 | 0.007 | 0.82 |
| NM_000436 | *OXCT1* | 3-oxoacid CoA transferase 1 | 3.51 | 0.019 | 1.00 |
| NM_001013257 | BCAM | basal cell adhesion molecule (Lutheran blood group) | 3.34 | 0.006 | 0.78 |
| NM_000290 | PGAM2 | phosphoglycerate mutase 2 (muscle) | 3.18 | 0.003 | 0.89 |
|  |  |  | DOWN |  |  |
| NM_006227 | *PLTP* | phospholipid transfer protein | 0.15 | 1.27E-07 | 0.75 |
| NM_004447 | *EPS8* | epidermal growth factor receptor pathway substrate 8 | 0.31 | 0.001 | 0.64 |
| NM_001935 | *DPP4* | dipeptidyl-peptidase 4 | 0.32 | 2.19E-05 | 0.86 |
| NM_001150 | *ANPEP* | alanyl (membrane) aminopeptidase | 0.34 | 2.60E-02 | 1.69 |
| NM_006332 | *IFI30* | interferon, gamma-inducible protein 30 | 0.35 | 0.006 | 0.33 |
| NM_005123 | *NR1H4* | nuclear receptor subfamily 1, group H, member 4 | 0.41 | 0.011 | 0.50 |
| NM_001008410 | *STEAP3* | STEAP family member 3, metalloreductase | 0.42 | 0.048 | 1.59 |
| NM_001547 | *IFIT2* | interferon-induced protein with tetratricopeptide repeats 2 | 0.43 | 0.040 | 0.42 |
| NM_015657 | *ABCA12* | ATP-binding cassette, sub-family A (ABC1), member 12 | 0.43 | 0.036 | 0.32 |
| NM_003028 | *SHB* | Src homology 2 domain containing adaptor protein B | 0.43 | 0.001 | 1.29 |
| Tem, temsirolimus; Veh, vehicle. Microarray analysis for KURC3 Tem tumors (Tem/P4/#1, #2, #3, and #4: n=4), KURC3 Veh tumors (Veh/P4/#1 and #2: n=2), KURC1 Tem tumors (Tem/P4/#1 and #2: n=2), and KURC1 Veh tumors (Veh/P4/#1: n=1). Top 10 upregulated and downregulated genes are listed (Student's *t*-test, *P*<0.05). | | | | | |

| **Table S6.** The number of probes for methylation and microarray analyses in the promotor region | | | |
| --- | --- | --- | --- |
| Promotor region |  |  |  |
| Methylation analysis |  | Microarray analysis |  |
| Hypomethylated | 4,263 (13.4%) | upregulated | 148 (3.5%) |
|  |  | downregulated | 95 (2.2%) |
|  |  | others | 4,020 (94.3%) |
| Hypermethylated | 1,534 (4.8%) | upregulated | 8 (0.5%) |
|  |  | downregulated | 29 (1.9%) |
|  |  | others | 1,497 (97.6%) |
| others | 25,936 (81.7%) | upregulated | 887 (3.4%) |
|  |  | downregulated | 639 (2.5%) |
|  |  | others | 24410 (94.1%) |
| Methylation analysis for KURC3 Tem/P4/#1 and Veh/P4/#1 tumors (each n=1). Microarray analysis for KURC3 Tem tumors (Tem/P4/#1, #2, #3, and #4: n=4), KURC3 Veh tumors (Veh/P4/#1 and #2: n=2), KURC1 Tem tumors (Tem/P4/#1 and #2: n=2), and KURC1 Veh tumors (Veh/P4/#1: n=1). Both hypomethylated (fold-change < 0.67) and upregulated (fold-change > 1.2 and Student's t-test, *P*<0.05) genes, and both hypermethylated (fold-change > 1.5) and downregulated (fold-change < 0.83 and Student's t-test, *P*<0.05) genes are listed. | | | |

| **Table S7.**  Methylation status and mRNA changes of mTOR signaling genes after temsirolimus treatment in temsirolimus-resistant (KURC3) or -sensitive (KURC1) xenograft tumors | | | | |
| --- | --- | --- | --- | --- |
| Gene | KURC3  Methylation  fold-change (Tem / Veh) | KURC3  mRNA  fold-change (Tem / Veh) | *P*-value | KURC1  mRNA fold-change (Tem / Veh) |
| *AKT1* | 0.92 | 0.86 | 0.35 | 1.20 |
| *AKT1S1* | 0.67 | 0.80 | 0.03 | 1.19 |
| *AKT2* | 0.81 | 0.98 | 0.55 | 1.04 |
| *AKT3* | 1.11 | 1.04 | 0.74 | 1.13 |
| *CAB39* | 1.36 | 0.99 | 0.97 | 0.95 |
| *CAB39L* | 3.35 | 0.99 | 0.94 | 0.95 |
| *CDC42* | 0.74 | 0.92 | 0.37 | 0.71 |
| *CHUK* | 1.34 | 0.98 | 0.44 | 0.88 |
| *DDIT4* | 0.84 | 1.14 | 0.27 | 1.40 |
| *DDIT4L* | 0.80 | 0.75 | 0.43 | 1.26 |
| *DEPTOR* | 0.74 | 0.93 | 0.15 | 0.65 |
| *EIF4B* | 1.02 | 1.20 | 0.20 | 1.22 |
| *EIF4E* | 0.58 | 0.97 | 0.81 | 1.12 |
| *EIF4EBP1* | 0.68 | 0.79 | 0.05 | 1.57 |
| *EIF4EBP2* | 0.68 | 1.01 | 0.84 | 0.79 |
| *FKBP1A* | 0.84 | N/A | N/A | N/A |
| *FKBP8* | 0.86 | 0.92 | 0.50 | 1.48 |
| *GSK3B* | 0.86 | 1.12 | 0.08 | 0.81 |
| *HIF1A* | 1.00 | 1.08 | 0.47 | 1.48 |
| *HRAS* | 0.74 | 1.04 | 0.56 | 1.48 |
| *HSPA4* | 0.75 | 0.92 | 0.40 | 0.84 |
| *IGF1* | 0.85 | 1.09 | 0.32 | 1.30 |
| *IGFBP3* | 0.91 | 0.44 | 0.05 | 1.96 |
| *IKBKB* | 0.68 | 1.17 | 0.14 | 1.03 |
| *ILK* | 0.99 | 1.02 | 0.78 | 0.86 |
| *INS* | 0.97 | 1.04 | 0.18 | 1.00 |
| *INSR* | 0.51 | 1.17 | 0.03 | 0.82 |
| *IRS1* | 1.19 | 0.99 | 0.96 | 1.24 |
| *MAPK1* | 1.18 | 1.01 | 0.90 | 0.96 |
| *MAPK3* | 0.89 | 0.91 | 0.02 | 1.20 |
| *MAPKAP1* | 0.87 | 1.29 | 0.08 | 1.01 |
| *MLST8* | 0.75 | 1.04 | 0.64 | 1.19 |
| *MTOR* | 0.87 | 0.97 | 0.69 | 1.05 |
| *MYO1C* | 0.89 | 0.97 | 0.85 | 0.95 |
| *PDPK1* | 0.88 | 1.05 | 0.34 | 0.87 |
| *PIK3CA* | 0.71 | 1.04 | 0.52 | 0.82 |
| *PIK3CB* | 0.99 | 0.92 | 0.57 | 0.81 |
| *PIK3CD* | 0.97 | 1.03 | 0.61 | 0.96 |
| *PIK3CG* | 0.98 | 1.02 | 0.16 | 0.87 |
| *PLD1* | 0.70 | 1.31 | 0.09 | 0.50 |
| *PLD2* | 0.88 | 0.99 | 0.98 | 0.99 |
| *PPP2CA* | 1.29 | 1.05 | 0.61 | 0.92 |
| *PPP2R2B* | 0.72 | 1.06 | 0.27 | 1.01 |
| *PPP2R4* | 0.99 | 0.81 | 0.28 | 0.89 |
| *PRKAA1* | 1.21 | 1.05 | 0.59 | 0.76 |
| *PRKAA2* | 0.60 | 1.13 | 0.12 | 0.79 |
| *PRKAB1* | 1.05 | 0.87 | 0.16 | 0.92 |
| *PRKAB2* | 1.06 | 1.20 | 0.07 | 0.69 |
| *PRKAG1* | 0.91 | 0.92 | 0.16 | 1.12 |
| *PRKAG2* | 0.98 | 0.86 | 0.12 | 1.24 |
| *PRKAG3* | 2.68 | 0.91 | 0.62 | 0.99 |
| *PRKCA* | 0.94 | 1.11 | 0.56 | 1.32 |
| *PRKCB* | 1.24 | 0.92 | 0.07 | 0.87 |
| *PRKCE* | 0.84 | 0.89 | 0.45 | 1.02 |
| *PRKCG* | 0.67 | 1.00 | 0.95 | 1.08 |
| *PTEN* | 0.93 | 1.34 | 0.10 | 1.00 |
| *RHEB* | 0.99 | 0.98 | 0.77 | 1.09 |
| *RHOA* | 0.72 | 1.37 | 0.16 | 1.11 |
| *RICTOR* | 0.69 | 0.99 | 0.97 | 0.92 |
| *RPS6* | 0.26 | 1.15 | 0.27 | 0.78 |
| *RPS6KA1* | 1.05 | 0.86 | 0.03 | 0.78 |
| *RPS6KA2* | 0.55 | 1.01 | 0.88 | 1.40 |
| *RPS6KA5* | 1.32 | 0.96 | 0.03 | 1.19 |
| *RPS6KB1* | 1.02 | N/A | N/A | N/A |
| *RPS6KB2* | 1.01 | 0.88 | 0.13 | 1.24 |
| *RPTOR* | 0.77 | 0.88 | 0.13 | 1.01 |
| *RRAGA* | 0.81 | 0.85 | 0.27 | 1.14 |
| *RRAGB* | 0.86 | 1.37 | 0.06 | 0.97 |
| *RRAGC* | 0.97 | 1.45 | 0.16 | 0.90 |
| *RRAGD* | 0.72 | 1.98 | 0.11 | 0.78 |
| *SGK1* | 1.18 | 0.84 | 0.16 | 1.15 |
| *STK11* | 0.99 | 0.97 | 0.63 | 1.11 |
| *STRADB* | 0.97 | N/A | N/A | N/A |
| *TELO2* | 1.08 | 0.91 | 0.39 | 1.09 |
| *TP53* | 0.91 | 0.96 | 0.78 | 0.70 |
| *TSC1* | 0.66 | 0.90 | 0.04 | 0.77 |
| *TSC2* | 0.92 | 1.01 | 0.85 | 1.06 |
| *ULK1* | 0.78 | 0.75 | 0.28 | 1.09 |
| *ULK2* | 0.89 | 0.98 | 0.76 | 0.83 |
| *VEGFA* | 1.02 | 1.25 | 0.03 | 1.85 |
| *VEGFB* | 0.98 | 1.18 | 0.03 | 1.21 |
| *VEGFC* | 0.46 | 0.89 | 0.20 | 1.11 |
| *YWHAQ* | 0.80 | 0.99 | 0.92 | 1.06 |
| Tem, temsirolimus; Veh, vehicle. Methylation analysis for KURC3 Tem/P4/#1 and Veh/P4/#1 tumors (each n=1). Microarray analysis for KURC3 Tem tumors (Tem/P4/#1, #2, #3, and #4: n=4), KURC3 Veh tumors (Veh/P4/#1 and #2: n=2), KURC1 Tem tumors (Tem/P4/#1 and #2: n=2), and KURC1 Veh tumors (Veh/P4/#1: n=1). N/A, not applicable | | | | |

| **Table S8.** mRNA changes after temsirolimus treatment in *WDSUB1, CPD*, *ANKRD12*, and *DNMT1* of temsirolimus-resistant (KURC3) or -sensitive (KURC1) xenograft tumors | | | | | |
| --- | --- | --- | --- | --- | --- |
|  |  |  | KURC3 | | KURC1 |
| Refseq | Gene | Gene description | mRNA fold-change (Tem / Veh) | *P*-value | mRNA fold-change (Tem / Veh) |
| NM_001128212 | *WDSUB1* | WD repeat, sterile alpha motif and U-box domain containing 1 | 1.25 | 0.135 | 0.85 |
| NM_001304 | *CPD* | carboxypeptidase D | 0.39 | 0.001 | 0.96 |
| NM_001083625 | *ANKRD12* | ankyrin repeat domain 12 | 0.62 | 0.004 | 1.02 |
| NM_001130823 | *DNMT1* | DNA (cytosine-5-)-methyltransferase 1 | 1.04 | 0.850 | 1.22 |
| Tem, temsirolimus; Veh, vehicle Microarray analysis for KURC3 Tem tumors (Tem/P4/#1, #2, #3, and #4: n=4), KURC3 Veh tumors (Veh/P4/#1 and #2: n=2), KURC1 Tem tumors (Tem/P4/#1 and #2: n=2), and KURC1 Veh tumors (Veh/P4/#1: n=1). | | | | | |
